# Supplementary material for: Microbubbles and UltraSound-accelerated Thrombolysis (MUST) for peripheral arterial occlusions: protocol for a phase II single-arm trial
Source: BMJ Open. 2017 Aug 11;7(8):e014365. doi: 10.1136/bmjopen-2016-014365 (PMC5724158; doi:10.1136/bmjopen-2016-014365)
Supplement: Author's manuscript [file bmjopen-2016-014365.draft_revisions.pdf]

BMJ Open

Microbubbles and UltraSound-accelerated Thrombolysis (MUST) for peripheral arterial occlusions: a phase-II single arm trial

Journal:	*BMJ Open*
Manuscript ID	bmjopen-2016-014365
Article Type:	Protocol
Date Submitted by the Author:	20-Sep-2016

Complete List of Authors:	Ebben, Harm; VU University Medical Center, Surgery<br>Nederhoed, Johanna; VU University Medical Center, Surgery<br>Lely, Rutger; VU University Medical Center, Radiology<br>Wisselink, Willem; VU University Medical Center, Surgery<br>Yeung, Kakkhee; VU University Medical Center, Surgery<br>MUST, Collaborators; VU University Medical Center, Surgery, Radiology, Cardiology, Physiology

**Primary Subject Heading**:	Surgery
Secondary Subject Heading:	Cardiovascular medicine, Surgery
Keywords:	peripheral arterial occlusion, thrombolysis, microbubbles, sonothrombolysis

Note: The following files were submitted by the author for peer review, but cannot be converted to PDF. You must view these files (e.g. movies) online.

Microbubbles in Vascular Surgery (Enhanced)-HD Ebben et al.mp4

SCHOLARONE™ Manuscripts

For peer review only

Microbubbles and UltraSound-accelerated Thrombolysis (MUST) for peripheral arterial occlusions: a phase-II single arm trial

Harm P. Ebben[1,4], Johanna H. Nederhoed[1], Rutger J. Lely[2], Willem Wisselink[1], Kak Khee Yeung[1,4] & MUST collaborators*

*Arjan W.J. Hoksbergen[1], Jan D. Blankensteijn[1], Bram B. van der Meijs[2], Martijn R. Meijerink[2], Otto Kamp[3], J. Slikkerveer[3], René J.P. Musters[4], Geert-Jan Tangelder[4]

Departments of [1]Surgery, [2]Radiology, [3]Cardiology and [4]Physiology, VU University Medical Center, Amsterdam, the Netherlands

Corresponding author

Dr. K.K. Yeung, MD, PhD

VU University Medical Center

P.O. Box 7057

1007 MB Amsterdam

The Netherlands

tel +31 (0)6 14 27 87 25

Key words: peripheral arterial occlusion, thrombolysis, microbubbles, ultrasound, sonothrombolysis

Word count: 2626 (*Excluding title page, abstract, references, figures and tables.)*

For peer review only

Abstract

Introduction

Acute peripheral arterial occlusions can be treated with intra-arterial catheter-directed thrombolysis as an alternative to surgical thromboembolectomy. Although less invasive, this treatment is time-consuming and carries a significant risk of hemorrhagic complications. Contrast-enhanced ultrasound using microbubbles could accelerate dissolution of thrombi by thrombolytic medications due to mechanical effects caused by oscillation; this could allow for lower dosages of thrombolytics and faster thrombolysis, thereby reducing the risk of hemorrhagic complications. In this study, the safety and practical applicability of this treatment will be investigated.

Methods and analysis

A single-arm phase-II trial will be performed in 20 patients with acute peripheral arterial occlusions eligible for thrombolytic treatment. Low-dose catheter-directed thrombolysis with urokinase will be used. The investigated treatment will be performed during the first hour of thrombolysis,consisting of intravenous infusion of 4 SonoVue vials (5 mL each, 20 mL total) of microbubbles with the use of local ultrasound at the site of occlusion. Primary endpoints are the incidence of complications and technical feasibility. Secondary endpoints are angiographic and clinical success, duration of thrombolytic infusion, treatment-related mortality, amputations, additional interventions, and quality of life.

Ethics and dissemination

Ethical approval for this study was obtained in 2015 from the Medical Ethics Committee (METC) of the VU University Medical Center, Amsterdam, the Netherlands. A statement of consent for this study was given by the Dutch national competent authority. Data will be presented at national and international conferences and published in a peer-reviewed journal.

Registration: Dutch National Trial Registry: NTR4731; European Clinical Trials Database (EudraCT) of the European Medicines Agency: 2014-003469-10.

For peer review only

ARTICLE FOCUS

- Protocol of a singe-arm phase-II trial to determine safety and technical feasibility of microbubble and ultrasound accelerated thrombolysis in patients with peripheral arterial occlusions.

KEY MESSAGES

- Catheter-directed thrombolysis is a less invasive alternative treatment to surgical thrombo-embolectomy in treatment of acute peripheral arterial occlusions but a major drawback of this treatment remains a significant risk of hemorrhagic complications.
- The concomitant application of microbubbles and ultrasound might both accelerate thrombolysis and allow for lower dosage of thrombolytics, thereby reducing the risk of hemorrhagic complications.

STRENGTHS AND LIMITATIONS

- This will be a first in man study to examine the safety and technical feasibility of therapeutic microbubbles, combined with the application of ultrasound and catheter-directed thrombolysis in peripheral arterial occlusions.
- This is a ‘single arm’ trial. The data will be used to inform a future large multicentre randomised controlled trial comparing conventional catheter-directed thrombolysis with microbubble and ultrasound accelerated thrombolysis

For peer review only

INTRODUCTION

Acute limb ischemia can be caused by a thrombus occluding an artery in an arm or leg. This is an emergency situation that can result in amputation or death if not treated successfully.[1] Intra-arterial infusion of thrombolytic agents, i.e. catheter-directed thrombolysis, can restore blood flow by dissolving the clot, as a less invasive alternative to surgical thromboembolectomy.[2] In comparison with the lysis of small arterial occlusions in patients with myocardial infarction, larger peripheral arterial occlusions require higher doses of lytic agents and infusion over a longer period of time. Inevitably, such treatment is accompanied by a risk of major hemorrhagic complications, such as hemorrhagic stroke, in up to 8% of patients.[3] Furthermore, this technique is time-consuming (several days of bed rest is usually required) and repeated angiography for treatment monitoring is needed, putting patients at risk for contrast-induced nephropathy. As a result, this leads to high morbidity rates and a significant patient burden. Methods to improve this therapy are therefore highly sought after.

A potential accelerator of thrombolytic therapy is contrast-enhanced ultrasound. Contrast agents, consisting of 5-10 µm gas-filled particles (microbubbles), have initially been used as diagnostic ultrasound contrast-enhancers. A new field of research investigates these agents for potential therapeutic purposes such as targeted drug delivery and thrombolysis.[4] The proposed mechanism of action in thrombolysis is that microbubbles can oscillate under the influence of ultrasound. At high intensities, this oscillation can lead to microbubble collapse and the production of mechanical forces on the clot surface, making the thrombus more susceptible to thrombolytics, thus accelerating thrombolysis.[5]

In early stages of clinical research, this technique has been shown to be efficient as treatment for acute cerebral stroke and acute myocardial.[6,7] Although the safety of their clinical administration in treating smaller arteries in the heart has been a topic of discussion in the past, post-marketing data for diagnostic indications showed continued safety after extensive research in more recent years.[8,9,10] For therapeutic thrombolytic purposes, this technique has been shown to be effective and safe in a porcine model of large peripheral arterial occlusions.[11] In this study, we will investigate the therapeutic application of microbubbles with ultrasound in combination with catheter-directed thrombolysis for patients with peripheral arterial occlusions. An illustrative video regarding our research project is available at https://vimeo.com/159929945/4aaf6770cf.

For peer review only

METHODS AND ANALYSIS

Study objectives

To investigate the safety and practical applicability of the therapeutic application of microbubbles and ultrasound in combination with catheter-directed thrombolysis for patients with peripheral arterial occlusions.

Design

The Microbubbles and UltraSound accelerated Thrombolysis (MUST) trial is a single-arm phase-II trial.

Primary study parameters

Main endpoints will be the safety and technical feasibility of the experimental treatment. Safety will be determined by treatment-related mortality, the occurrence of Adverse Events (AEs), Serious Adverse Events (SAEs) and Suspected Unexpected Serious Adverse Reactions (SUSARs). AEs will be defined as any undesirable experience occurring to a subject during the experimental treatment period, whether or not considered to be related to the investigational drug or intervention. SAEs will be defined as any untoward medical occurrence or effect that at any dose results in death; is life threatening (at the time of the event); requires hospitalization or prolongation of existing in-patients' hospitalization; results in persistent or significant disability or incapacity; is a new event of the trial likely to affect the safety of the subjects, such as an unexpected outcome of an adverse reaction. SUSARs, which are related to the microbubble infusion and ultrasound application, are the formation of micro embolisms resulting in occlusion of the microcirculation, hemorrhages, hypotension, heart rhythm disorders and anaphylaxis. See the paragraph *adverse events* for detailed handling procedures of AEs, SAEs and SUSARs. Hemorrhagic complications related to thrombolytic therapy will be reported according the Standardized Bleeding Definitions for Cardiovascular Clinical Trials proposed by Mehran et al.[12]
Technical feasibility will be defined as accomplishment of the experimental protocol during the first hour of thrombolysis.

Secondary study parameters

Angiographic success will be defined as dissolution of >95% of the thrombus with outflow to at least 1 crural arteryClinical change/success will be reported according to Rutherford's recommended scale for gauging changes in clinical status (Table I). Amputations will be defined as either major (above or below knee amputation) or minor (metatarsal or toe amputation). Additional interventions will be categorized as either surgical (for example thromboembolectomy, bypass graft surgery) or percutaneous (Percutaneous Transluminal Angioplasty, stent placement) and as either required for restoration of patency or necessary for correction of underlying lesions. We will also determine microcirculation of the limb (by Laser Doppler measurements, Perimed Instruments, Järfälla, Sweden), 30-day mortality, conversion to surgery, serum fibrinogen concentrations measured during thrombolytic treatment on a daily basis, pain by Visual Analogue Scale (VAS) and quality of life by SF-36 questionnaires. The duration of thrombolysis will be defined by the time-span between initiation and completion angiography.

Table I: *Rutherford's recommended scale for gauging changes in clinical status*

Adapted from: Rutherford RB, Baker JD, Ernst C, Johnston KW, Porter JM, Ahn S, et al. Recommended standards for reports dealing with lower extremity ischemia: revised version. J Vasc Surg. 1997;26(3):517–38.

+3	Markedly improved: No ischemic symptoms, and any foot lesions completely healed; ABI essentially "normalized" (increased to more than 0.90)
+2	Moderately improved: No open foot lesions; still symptomatic but only with exercise and improved by at least one clinical chronic ischemia category; ABI not normalized but increased by more than 0.10
+1	Minimally improved: Greater than 0.10 increase in ABI* but no categorical improvement or vice versa (i.e., upward categorical shift without an increase in ABI of more than 0.10)
0	No change: No categorical shift and less than 0.10 change in ABI

For peer review only

−1	Mildly worse: No categorical shift but ABI decreased more than 0.10, or downward categorical shift with ABI decrease less than 0.10
−2	Moderately worse: One category worse or unexpected minor amputation
−3	Markedly worse: More than one category worse or unexpected major amputations
*In cases where the ABI cannot be accurately measured, an index based on the toe pressure, or any measurable pressure distal to the site of revascularization, may be substituted.	

Abbreviations: *ABI=Ankle Brachial Index*

Patients and eligibility criteria

In this phase-II trial, a total of 20 patients with acute lower limb ischemia proven by angiography will be included. Eligibility criteria are listed in Table II.

Table II: *Eligibility criteria*

Inclusion criteria	• Men and women older than 18 and younger than 85 years old<br>• Patients with a maximum of 2 weeks of symptoms for lower limb ischemia due to thrombosed/occluded femoropopliteal or femorocrural native arteries or femoropopliteal or femorocrural venous or prosthetic bypass grafts<br>• Patients appropriate for thrombolysis i.e. with acute lower limb ischemia class I and IIa according to the Rutherford classification<br>• Patients who understand the nature of the procedure and provide written informed consent before enrollment in the study
Exclusion criteria	• Patients with clinical complaints of acute lower limb ischemia due to thrombosis of femoropopliteal or femorocrural native arteries, or femoropopliteal or femorocrural venous or prosthetic bypass grafts for more than 2 weeks<br>• Patients with absolute contraindications for administration of antiplatelet therapy, anticoagulants or thrombolytics<br>• History of recent (less than 6 weeks) ischemic stroke, cerebral hemorrhagic or myocardial infarction<br>• Patients with recent (less than 6 weeks) surgery<br>• Severe hypertension (diastolic blood pressure greater than 110 mm Hg, systolic blood pressure higher than 200 mm Hg)<br>• Current malignancy or severe co-morbid condition with a life expectancy of less than 6 months<br>• Patients with uncorrected bleeding disorders (gastrointestinal ulcer, menorrhagia, liver failure)<br>• Women with childbearing potential not taking adequate contraceptives or currently breastfeeding<br>• Pregnancy<br>• Patients who are currently participating in another investigational drug or device study<br>• Patients younger than 18 years or older than 85 years<br>• Patients with contraindications for Sonovue microbubbles i.e.:<br>○ Hypersensitivity to sulphur hexafluoride or to any of the components of SonoVue<br>○ Recent acute coronary syndrome or clinically unstable ischemic cardiacdisease, including: evolving or ongoing myocardial infarction, unstable angina at rest within the last 7 days, significant worsening of cardiac symptoms within the last 7 days, recent coronary artery intervention or other factors suggesting clinical instability (for example, recent

For peer review only

	deterioration of ECG, laboratory or clinical findings), acute cardiac failure, Class III/IV cardiac failure, or severe rhythm disorders<br>○ Patients known to have right-to-left cardiac shunts, severe pulmonary hypertension (pulmonary artery pressure >90 mmHg), uncontrolled systemic hypertension, or adult respiratory distress syndrome<br>○ Patients with cardiovascular instability where dobutamine is contraindicated

Data handling

We will keep an electronic log of patients who fulfill the eligibility criteria, patients who are invited to participate in the study, patients recruited and patients who withdraw from the study. Reasons for non-recruitment will also be recorded. We will attempt to collect reasons for non-participation from patients who decline to take part. During the course of the study, we will document reasons for withdrawal from the study and loss to follow-up. Data will be stored electronically in Case Report Forms by using *Open Clinica* software with audit trail functionality. Only anonymized information will be stored and participants will only be identifiable by their unique study number, which will be kept in a separate file. Data will be securely stored on Open Clinica servers for 15 years according to national guidelines.

Study procedures

Intervention

A flow chart of the patient work-up after presentation in our hospital is presented in Figure 1. Low-dose thrombolytic treatment with urokinase will be initiated following our standard institutional protocol: a catheter is placed intra-arterially in the affected artery and a bolus injection of 500,000 International Units (IU) of urokinase (Medacinase Urokinase, Medac GmbH, Hamburg, Germany) will be followed by the continuous infusion of 50,000 units of UK per hour and 9,600 IU of heparin per 24 hours. The experimental treatment consists of (in addition to the standard thrombolytic therapy) the use of local ultrasound (Philips iE33 Ultrasound Machine, Eindhoven, the Netherlands), and the intravenous infusion of 4 vials (5 mL each, total 20 mL) of microbubbles (SonoVue, Bracco Imaging Europe B.V., the Netherlands) during the first hour of thrombolysis with urokinase. An ACIST VueJect (Bracco Imaging Europe B.V., the Netherlands) infusion pump will be used to infuse the 4 vials continuously. Ultrasound will be intermittently activated (3 seconds manual flash to burst microbubbles with Mechanical Index (MI) 1.08 (pulse duration 20 microseconds, frequency 1.8 Mhz, framerate 39 Hz), 7 seconds of visualization of inflow of the microbubbles at MI±0.11, at the site of occlusion during the first hour of thrombolysis.

For peer review only

Figure 1: *Flow chart of patient work-up after presentation*

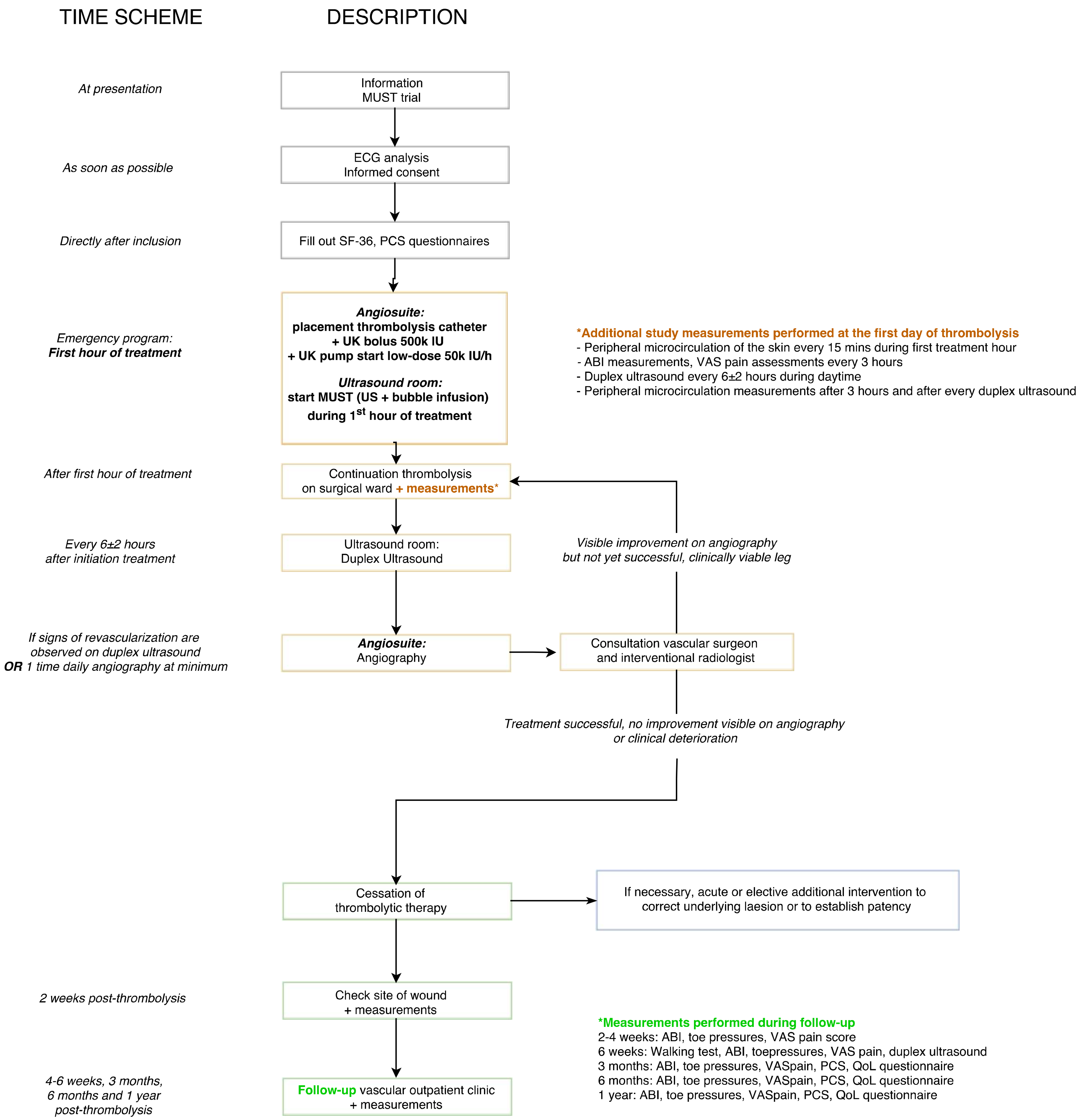

For peer review only - http://bmjopen.bmj.com/site/about/guidelines.xhtml

Abbreviations: *ECG = Electro Cardiography, UK = urokinase, US = UltraSound, ABI = Ankle Brachial Index, VAS = Visual Analogue Scale, PCS= Catastrophizing Scale, QoL=Quality of Life.*

Assessments

Diagnostic measurements

Additional daily diagnostic measurements during including ECG, duplex ultrasound, angiography and microcirculation measurements (by Laser Doppler flowmetry) will be performed as depicted in Figure 1.

A duplex ultrasound will be performed every 6±2 hours to monitor for signs of revascularization. When resumption of flow is visualized by duplex ultrasound, angiography will be performed to confirm flow. Angiography will be performed at least once daily as standard procedure. Outside of routine hospital working hours, angiography will only be performed in emergencies as per standard care.

A standardized pain score (Visual Analogue Scale, 1-10), and Pain Catastrophizing Scale will be recorded every 3 hours by a nurse practitioner, research fellow or surgical resident to assess pain.

Fibrinogen monitoring

Following our standard institutional thrombolysis protocol, fibrinogen concentration will be checked during thrombolysis with the following criteria for treatment modification: If <1.0 g/L, the urokinase infusion rate will be lowered to 25,000 IU/h; if <0.5 g/L, thrombolysis must be aborted temporarily and replaced by normal saline infusion. Three hours following treatment modifications, fibrinogen concentration will be reevaluated and when >1.0 g/L thrombolysis will be restarted at an initial low dose urokinase of 50,000 IU/h.

Post-procedural anticoagulation

After successful thrombolysis, the patient will be heparinized with low-molecular weight heparin (fraxiparine) dosed based on body weight: <50 kg: 2 times a day 3,800 IU (= 0.4 mL), 50-80 kg: 2 times a day 5,700 IU (= 0.6 mL), >80 kg: 2 times a day 7,600 IU (= 0.8 mL).

Concomitant therapy with coumarin derivatives will also be started at that time. Activated partial thromboplastin time (aPTT) will be measured daily during heparin treatment. The target range international normalized ratio (INR) will be 2.5 to 3.5; if this value is reached, heparinization will be stopped and coumarin treatment will be continued.

Follow-up

Outpatient follow-up will take place at specific time points for a total duration of 1 year, measurements performed during follow-up visits are depicted in Figure 1.

Adverse events

Adverse events will be recorded in detail in the electronic patient record. Any serious adverse events that occur after joining the trial will be reported to the accredited Medical Ethics Committee of our institution according to national and institutional guidelines. All adverse events will be followed up until they have abated, or until a stable situation has been reached. Depending on the event, follow up may require additional tests or medical procedures as indicated, and/or referral to a general physician or medical specialist.

Statistical analysis

Categorized epidemiologic/descriptive patient variables are summarized with frequencies and will be analyzed with Fischer's exact test or the Pearson Chi-squared test. To avoid possible violations of the assumptions for parametric testing, such as a normal distribution pattern, we will employ non-parametric methods such as a Spearman rank correlation and a Mann-Whitney U test in the case of a skewed distribution or log transformation. For associations of two outcome measurements, we will use a correlation (Spearman rank) or single regression analysis. We will analyze the following outcomes by means of Kaplan-Meier curves: patency rate, amputation-free rate, and intervention-free rate. We will assess heterogeneity in prognostic factors as a secondary analysis by means of Chi-squared tests. All tests will be performed two-sided, and a $p<0.05$ will be considered to be statistically significant.

For peer review only

ETHICS AND DISSEMINATION

The study will be conducted according to the principles of the Declaration of Helsinki (Brazil, October 2013), and in accordance with the Medical Research Involving Human Subjects Act (WMO). An Investigator Site File will be produced in advance of the study conforming to institutional guidelines. Furthermore we will create Case Report Forms by using *Open Clinica* software, GCP and 21 CFR Part 11 compliant, to handle patient data.

The study has been registered in the Dutch Trial Register, at the Dutch National Central Committee on Research Involving Human Subjects (CCMO) and in the European Clinical Trials Database (EudraCT) of the European Medicines Agency. The results of this study will be submitted for publication in a peer-reviewed journal, regardless of the outcome of this study, according to the CCMO statement on publication policy. Data will also be presented at international conferences.

For peer review only

DISCUSSION

The Microbubbles and UltraSound-accelerated Thrombolysis (MUST) trial is a phase-II single-arm clinical trial. In this study the safety and feasibility of an experimental ultrasound technique will be investigated for the first time in patients with large peripheral arterial occlusions.

We believe that this procedure is safe and can accelerate thrombolysis,thereby allowing for reduction of thrombolytic dosage, which in turn reduces the risk of major hemorrhagic complications.

An experimental bolus therapy with microbubbles and ultrasound could accelerate thrombolysis because at high ultrasound intensities microbubbles can collapse, resulting in mechanical forces on the clot surface. The formation of small channels in the thrombus lead to exposure of a larger total surface susceptible to thrombolytics.[5]

In regard to the therapeutic application of contrast agents, several studies have been performed in patients with ischemic stroke and myocardial infarction. A systematic review of sonothrombolysis shows that this treatment option improves clinical and long-term outcomes, while potentially reducing bleeding risk, in patients with ischemic stroke.[13] Nevertheless, dose escalation studies show that the safety (in terms of bleeding and micro emboli) needs to be further investigated before enrolling patients in phase-III trials.[14] Few and heterogeneous studies examined the therapeutic application of sonothrombolysis in patients with myocardial infarction. Although pilot studies affirm safety and feasibility, the application of therapeutic ultrasound with longer pulse durations (20 microseconds vs. 5 microseconds) was reported to result in unexpected coronary vasoconstriction in a recent clinical trial.[15]
Potential reported mechanisms for this effect are the summative effect of myocardial ischemia, reperfusion damage and long-pulse-duration sonoporation on endothelial damage, all leading to calcium overload. However, patients with peripheral arterial occlusions are mostly chronic vascular patients who often have received previous treatments in the respective artery, for example thrombolytic therapy, percutaneous transluminal angioplasty, thromboembolectomy or bypass surgery. The mechanical manipulation of the vascular wall during all these treatments is extensive. Furthermore, during standard thrombolytic treatment, arteries are manipulated and perforated on purpose to insert guide wires and catheters. Hence, vascular spasms during these peripheral treatments are normal and non-threatening to the patient, in contrast to spasms in small coronary arteries.

The administration of ultrasound contrast agents has been accompanied by important discussions regarding safety concerns in the past[8,16]. As a response to the occurrence of SAEs, the US Food and Drug Administration (FDA) issued a labeling change and warnings for contrast agents in 2007. Consequently, new studies on the risks of contrast agents were performed and these established their safety.[17] In Europe the European Medicines Agency conducted an extensive evaluation of the contrast agent SonoVue and concluded with the decision to remove its previously instated contraindications.[18]

In regard to SonoVue contrast agent dose regimens, the maximum approved dose for diagnostic indications is 4.8 mL injected over a short time-period.[19] For therapeutic purposes, we anticipate a higher total dose of 20 mL in one hour. In an 80 kg patient this equates to 0.25 mL/kg. Clinical studies have reported cumulative doses up to 0.6 mL/kg of SonoVue contrast agent without the occurrence of adverse events or side effects.[20] During the experimental protocol with microbubble infusion, patients will be continuously monitored.

As with all contrast agents, the risk of anaphylactic reactions to contrast remains. Therefore, administration of contrast agents in a center with full resuscitation possibilities is mandatory. Furthermore, during the first hours of administration, monitoring of vital parameters of patients is important.

In this study, thrombolysis is performed with the fibrinolytic urokinase, which is the most used fibrinolytic agent for the treatment of peripheral arterial occlusions worldwide and is standard care in the Netherlands. Some countries use tissue plasminogen activator for this indication. A Cochrane review on the topic states that there is no evidence that (r)t-PA is more effective than urokinase for patients with peripheral arterial occlusion.[21]

If the application of microbubbles and ultrasound concomitant to catheter-directed thrombolysis is shown to be safe and technically feasible based on this phase-II trial, we anticipate a funding application for a larger randomized controlled trial with a comparative group to assess and compare efficacy of this treatment.

For peer review only

Although the efficacy of the currently described protocol cannot be adequately compared within this study design, we will discuss the outcomes relative to a historic control group that had previously received our standard hospital thrombolysis protocol.[22]

Successful thrombolysis is strongly predictive of amputation-free survival with vascular patency for at least one year.[23] A longer duration of thrombolysis inevitably exposes a patient to a higher thrombolytic dose and higher risk of hemorrhage, in addition to an already increased patient burden because of prolonged bed rest. Therefore ultimately, acceleration of thrombolysis with microbubbles could benefit the patient because of a shorter therapy time, a lower risk of hemorrhagic complications and a decrease in patient burden.

ACKNOWLEDGEMENTS:
We would like to thank Laura van Wieringen, Lloyd Belliot, Joyce Lu, Jorn Meekel, Ted van Schaik and Sean Matheiken for their assistance in preparation of this study.

AUTHOR CONTRIBUTIONS

HE, JN, WW and KK planned and designed the research, HE wrote the manuscript, HE, JN, RL and KK critically revised the manuscript and all authors and MUST collaborators approved the final version of the manuscript.

FUNDING: This study is supported by the Departments of Surgery, Radiology, Cardiology and Physiology of the VU University Medical Center Amsterdam, the Netherlands and by a research grant from LamePro B.V., the Netherlands.

COMPETING INTERESTS: none

For peer review only

REFERENCES

1. Creager, M. A., Kaufman, J. A., & Conte, M. S. Clinical practice. Acute limb ischemia. *N Engl J Med*, 2012. 366(23), 2198–2206.
2. Berridge, D. C., Kessel, D., & Robertson, I. Surgery versus thrombolysis for acute limb ischaemia: initial management. *Cochrane Database Syst Rev*, (3) (2013), CD002784.
3. van den Berg, J. C. Thrombolysis for acute arterial occlusion. *J Vasc Surg*, 2009. 52(2), 512–515.
4. Dijkmans, P. A., Juffermans, L. J. M., Musters, R. J. P., Wamel, A. Van, Cate, F. J., Gilst, W. Van, Kamp, O. Microbubbles and ultrasound: from diagnosis to therapy. *Eur J Echocardiogr.* 2004 Aug;5(4):245-56.
5. Everbach, E. C., & Francis, C. W. (2000). Cavitational mechanisms in ultrasound-accelerated thrombolysis at 1 MHz. *Ultrasound Med Biol*, 2000 26(7), 1153–1160.
6. Balucani C, Alexandrov A V. Ultrasound- and microspheres-enhanced thrombolysis for stroke treatment: state of the art. *Curr Cardiol Rep*. 2010;12(1):34–41.
7. Slikkerveer J, Kleijn SA, Appelman Y, Porter TR, Veen G, van Rossum AC, et al. Ultrasound enhanced prehospital thrombolysis using microbubbles infusion in patients with acute ST elevation myocardial infarction: pilot of the Sonolysis study. *Ultrasound Med Biol*. 2012;38(2):247–52.
8. Dijkmans PA, Visser CA, Kamp O. Adverse reactions to ultrasound contrast agents: Is the risk worth the benefit? *Eur J Echocardiogr.* 2005 *Oct;6(5):363-6.*
9. Roos ST, Juffermans LJM, van Royen N, van Rossum AC, Xie F, Appelman Y, et al. Unexpected High Incidence of Coronary Vasoconstriction in the Reduction of Microvascular Injury Using Sonolysis (ROMIUS) Trial. *Ultrasound Med Biol*. 2016 May 5 (Epub ahead of print).
10. Appis a. W, Tracy MJ, Feinstein SB. Update on the safety and efficacy of commercial ultrasound contrast agents in cardiac applications. Echo Res Pract. 2015;2(2):R55–62.
11. Ebben HP, Nederhoed JH, Slikkerveer J, Kamp O, Tangelder GWJM, Musters RJP, et al. Therapeutic application of contrast-enhanced ultrasound and low-dose urokinase for thrombolysis in a porcine model of acute peripheral arterial occlusion. *J Vasc Surg*. 2015 Aug;62(2):477–85.
12. Mehran R, Rao S V., Bhatt DL, Gibson CM, Caixeta A, Eikelboom J, et al. Standardized Bleeding Definitions for Cardiovascular Clinical Trials: A Consensus Report From the Bleeding Academic Research Consortium. *Circulation*. 2011 Jun 13;123(23):2736–47.
13. Roos ST, Juffermans LJM, Slikkerveer J, Unger EC, Porter TR, Kamp O. Sonothrombolysis in acute stroke and myocardial infarction: A systematic review. *IJC Hear Vessel*.; 2014;4:1–6.
14. Molina CA, Barreto AD, Tsivgoulis G, Sierzenski P, Malkoff MD, Rubiera M, et al. Transcranial ultrasound in clinical sonothrombolysis (TUCSON) trial. *Ann Neurol*.; 2009;66(1):28–38.
15. Roos ST, Juffermans LJM, van Royen N, van Rossum AC, Xie F, Appelman Y, et al. Unexpected High Incidence of Coronary Vasoconstriction in the Reduction of Microvascular Injury Using Sonolysis (ROMIUS) Trial. *Ultrasound Med Biol* [Internet]. 2016;1–10.
16. Douglas PS, Weyman AE, Lindner JR, Wei K.Contrast echocardiography: past, present, and...future? *JACC Cardiovasc Imaging.* 2008 Jan;1(1):107-10.
17. FDA Advisory Committee Briefing Document Cardiovascular and Renal Drugs Advisory Committee June 24th 2008. (Accessed on 23-05-2016, available at http://www.fda.gov/ohrms/dockets/ac/08/briefing/2008- 4369b1-01.pdf).
18. Sonovue: EPAR-Assessment Report-Variation. (Accessed on 23-05-2016, available at http://www.ema.europa.eu/docs/en_GB/document_library/EPAR_-_Assessment_Report_-_Variation/human/000303/WC500170218.pdf)
19. EMA Sonovue Summary of Product Characteristics (SmPC) (Accessed on 23-05-2016, available at http://www.ema.europa.eu/docs/en_GB/document_library/EPAR_-_Product_Information/human/000303/WC500055380.pdf)
20. Bokor D, Chambers JB, Rees PJ, Mant TG, Luzzani F, Spinazzi A. Clinical safety of SonoVue, a new contrast agent for ultrasound imaging, in healthy volunteers and in patients with chronic obstructive pulmonary disease. *Invest Radiol*.; 2001;36(2):104–9.
21. Robertson I, Kessel DO, Berridge DC. Fibrinolytic agents for peripheral arterial occlusion. *Cochrane Database Syst Rev*. 2013 Dec 19;12:CD001099.
22. Ebben HP, Nederhoed JH, Lely RJ, Meijerink MR, van der Meijs BB, Wisselink W, et al. Low-dose Thrombolysis for Thromboembolic Lower Extremity Arterial Occlusions is Effective Without Major Hemorrhagic Complications. *Eur J Vasc Endovasc Surg*. 2014 Nov;48(5):551-8.

For peer review only

23. G. Plate, S. Oredsson, J. Lanke. When is thrombolysis for acute lower limb ischemia worthwhile? *Eur J Vasc Endovasc Surg*, 37 (2) (2009), pp. 206–212

For peer review only

SPIRIT

STANDARD PROTOCOL ITEMS: RECOMMENDATIONS FOR INTERVENTIONAL TRIALS

SPIRIT 2013 Checklist: Recommended items to address in a clinical trial protocol and related documents*

BOLD AND ITALIC TEXT MEANS CHECKED

Section/item	Item No	Description
**Administrative information**		
Title	***1***	***Descriptive title identifying the study design, population, interventions, and, if applicable, trial acronym***
Trial registration	***2a***	***Trial identifier and registry name. If not yet registered, name of intended registry***
	2b	***All items from the World Health Organization Trial Registration Data Set***
Protocol version	***3***	***Date and version identifier***
Funding	***4***	***Sources and types of financial, material, and other support***
Roles and responsibilities	***5a***	***Names, affiliations, and roles of protocol contributors***
	5b	***Name and contact information for the trial sponsor***
	5c	***Role of study sponsor and funders, if any, in study design; collection, management, analysis, and interpretation of data; writing of the report; and the decision to submit the report for publication, including whether they will have ultimate authority over any of these activities***
	5d	***Composition, roles, and responsibilities of the coordinating centre, steering committee, endpoint adjudication committee, data management team, and other individuals or groups overseeing the trial, if applicable (see Item 21a for data monitoring committee)***
**Introduction**		
Background and rationale	***6a***	***Description of research question and justification for undertaking the trial, including summary of relevant studies (published and unpublished) examining benefits and harms for each intervention***
	6b	***Explanation for choice of comparators → NOT APPLICABLE***

For peer review only

Objectives	*7*	*Specific objectives or hypotheses*
Trial design	*8*	*Description of trial design including type of trial (eg, parallel group, crossover, factorial, single group), allocation ratio, and framework (eg, superiority, equivalence, noninferiority, exploratory)*

Methods: Participants, interventions, and outcomes

Study setting	*9*	*Description of study settings (eg, community clinic, academic hospital) and list of countries where data will be collected. Reference to where list of study sites can be obtained*
Eligibility criteria	*10*	*Inclusion and exclusion criteria for participants. If applicable, eligibility criteria for study centres and individuals who will perform the interventions (eg, surgeons, psychotherapists)*
Interventions	*11a*	*Interventions for each group with sufficient detail to allow replication, including how and when they will be administered*
	11b	*Criteria for discontinuing or modifying allocated interventions for a given trial participant (eg, drug dose change in response to harms, participant request, or improving/worsening disease)*
	11c	*Strategies to improve adherence to intervention protocols, and any procedures for monitoring adherence (eg, drug tablet return, laboratory tests)*
	11d	*Relevant concomitant care and interventions that are permitted or prohibited during the trial*
Outcomes	*12*	*Primary, secondary, and other outcomes, including the specific measurement variable (eg, systolic blood pressure), analysis metric (eg, change from baseline, final value, time to event), method of aggregation (eg, median, proportion), and time point for each outcome. Explanation of the clinical relevance of chosen efficacy and harm outcomes is strongly recommended*
Participant timeline	*13*	*Time schedule of enrolment, interventions (including any run-ins and washouts), assessments, and visits for participants. A schematic diagram is highly recommended (see Figure)*
Sample size	*14*	*Estimated number of participants needed to achieve study objectives and how it was determined, including clinical and statistical assumptions supporting any sample size calculations*
Recruitment	*15*	*Strategies for achieving adequate participant enrolment to reach target sample size*

Methods: Assignment of interventions (for controlled trials)

Allocation:

Sequence generation	*16a*	*Method of generating the allocation sequence (eg, computer-generated random numbers), and list of any factors for stratification. To reduce predictability of a random sequence, details of any planned restriction (eg, blocking) should be provided in a separate document that is unavailable to those who enrol participants or assign interventions*
Allocation concealment mechanism	*16b*	*Mechanism of implementing the allocation sequence (eg, central telephone; sequentially numbered, opaque, sealed envelopes), describing any steps to conceal the sequence until interventions are assigned*
Implementation	*16c*	*Who will generate the allocation sequence, who will enrol participants, and who will assign participants to interventions*
Blinding (masking)	*17a*	*Who will be blinded after assignment to interventions (eg, trial participants, care providers, outcome assessors, data analysts), and how*
	17b	*If blinded, circumstances under which unblinding is permissible, and procedure for revealing a participant's allocated intervention during the trial*
**Methods: Data collection, management, and analysis**		
Data collection methods	*18a*	*Plans for assessment and collection of outcome, baseline, and other trial data, including any related processes to promote data quality (eg, duplicate measurements, training of assessors) and a description of study instruments (eg, questionnaires, laboratory tests) along with their reliability and validity, if known. Reference to where data collection forms can be found, if not in the protocol*
	18b	*Plans to promote participant retention and complete follow-up, including list of any outcome data to be collected for participants who discontinue or deviate from intervention protocols*
Data management	*19*	*Plans for data entry, coding, security, and storage, including any related processes to promote data quality (eg, double data entry; range checks for data values). Reference to where details of data management procedures can be found, if not in the protocol*
Statistical methods	*20a*	*Statistical methods for analysing primary and secondary outcomes. Reference to where other details of the statistical analysis plan can be found, if not in the protocol*
	20b	*Methods for any additional analyses (eg, subgroup and adjusted analyses)*
	20c	*Definition of analysis population relating to protocol non-adherence (eg, as randomised analysis), and any statistical methods to handle missing data (eg, multiple imputation)*

Methods: Monitoring		
Data monitoring	***21a***	***Composition of data monitoring committee (DMC); summary of its role and reporting structure; statement of whether it is independent from the sponsor and competing interests; and reference to where further details about its charter can be found, if not in the protocol. Alternatively, an explanation of why a DMC is not needed***
	21b	***Description of any interim analyses and stopping guidelines, including who will have access to these interim results and make the final decision to terminate the trial***
**Harms**	**22**	**Plans for collecting, assessing, reporting, and managing solicited and spontaneously reported adverse events and other unintended effects of trial interventions or trial conduct**
Auditing	***23***	***Frequency and procedures for auditing trial conduct, if any, and whether the process will be independent from investigators and the sponsor***
**Ethics and dissemination**		
Research ethics approval	***24***	***Plans for seeking research ethics committee/institutional review board (REC/IRB) approval***
Protocol amendments	***25***	***Plans for communicating important protocol modifications (eg, changes to eligibility criteria, outcomes, analyses) to relevant parties (eg, investigators, REC/IRBs, trial participants, trial registries, journals, regulators)***
Consent or assent	***26a***	***Who will obtain informed consent or assent from potential trial participants or authorised surrogates, and how (see Item 32)***
	26b	***Additional consent provisions for collection and use of participant data and biological specimens in ancillary studies, if applicable***
Confidentiality	***27***	***How personal information about potential and enrolled participants will be collected, shared, and maintained in order to protect confidentiality before, during, and after the trial***
Declaration of interests	***28***	***Financial and other competing interests for principal investigators for the overall trial and each study site***
Access to data	***29***	***Statement of who will have access to the final trial dataset, and disclosure of contractual agreements that limit such access for investigators***
Ancillary and post-trial care	***30***	***Provisions, if any, for ancillary and post-trial care, and for compensation to those who suffer harm from trial participation***

For peer review only

Dissemination policy	***31a***	***Plans for investigators and sponsor to communicate trial results to participants, healthcare professionals, the public, and other relevant groups (eg, via publication, reporting in results databases, or other data sharing arrangements), including any publication restrictions***
	31b	***Authorship eligibility guidelines and any intended use of professional writers***
	31c	***Plans, if any, for granting public access to the full protocol, participant-level dataset, and statistical code***
**Appendices**		
Informed consent materials	***32***	***Model consent form and other related documentation given to participants and authorised surrogates***
Biological specimens	***33***	***Plans for collection, laboratory evaluation, and storage of biological specimens for genetic or molecular analysis in the current trial and for future use in ancillary studies, if applicable***

*It is strongly recommended that this checklist be read in conjunction with the SPIRIT 2013 Explanation & Elaboration for important clarification on the items. Amendments to the protocol should be tracked and dated. The SPIRIT checklist is copyrighted by the SPIRIT Group under the Creative Commons "Attribution-NonCommercial-NoDerivs 3.0 Unported" license.

For peer review only

BMJ Open

Microbubbles and UltraSound-accelerated Thrombolysis (MUST) for peripheral arterial occlusions: protocol for a phase-II single arm trial

Journal:	*BMJ Open*
Manuscript ID	bmjopen-2016-014365.R1
Article Type:	Protocol
Date Submitted by the Author:	12-Jan-2017
Complete List of Authors:	Ebben, Harm; VU University Medical Center, Surgery<br>Nederhoed, Johanna; VU University Medical Center, Surgery<br>Lely, Rutger; VU University Medical Center, Radiology<br>Wisselink, Willem; VU University Medical Center, Surgery<br>Yeung, Kakkhee; VU University Medical Center, Surgery<br>MUST, Collaborators; VU University Medical Center, Surgery, Radiology, Cardiology, Physiology
**Primary Subject Heading**:	Surgery
Secondary Subject Heading:	Cardiovascular medicine, Surgery
Keywords:	peripheral arterial occlusion, thrombolysis, microbubbles, sonothrombolysis

Note: The following files were submitted by the author for peer review, but cannot be converted to PDF. You must view these files (e.g. movies) online.

Microbubbles in Vascular Surgery (Enhanced)-HD Ebben et al.mp4

SCHOLARONE™
Manuscripts

Microbubbles and UltraSound-accelerated Thrombolysis (MUST) for peripheral arterial occlusions: protocol for a phase-II single arm trial

Harm P. Ebben[1,4], Johanna H. Nederhoed[1], Rutger J. Lely[2], Willem Wisselink[1], Kak Khee Yeung[1,4] & MUST collaborators*

*Arjan W.J. Hoksbergen[1], Jan D. Blankensteijn[1], Bram B. van der Meijs[2], Martijn R. Meijerink[2], Otto Kamp[3], J. Slikkerveer[3], René J.P. Musters[4], Geert-Jan Tangelder[4]

Departments of [1]Surgery, [2]Radiology, [3]Cardiology and [4]Physiology, VU University Medical Center, Amsterdam, the Netherlands

Corresponding author

Dr. K.K. Yeung, MD, PhD
VU University Medical Center
P.O. Box 7057
1007 MB Amsterdam
The Netherlands
tel +31 (0)6 14 27 87 25

Key words: peripheral arterial occlusion, thrombolysis, microbubbles, ultrasound, sonothrombolysis

Word count: 2626 (*Excluding title page, abstract, references, figures and tables.)*

Protocol version January 2017

For peer review only

Abstract

Introduction

Acute peripheral arterial occlusions can be treated with intra-arterial catheter-directed thrombolysis as an alternative to surgical thromboembolectomy. Although less invasive, this treatment is time-consuming and carries a significant risk of hemorrhagic complications. Contrast-enhanced ultrasound using microbubbles could accelerate dissolution of thrombi by thrombolytic medications due to mechanical effects caused by oscillation; this could allow for lower dosages of thrombolytics and faster thrombolysis, thereby reducing the risk of hemorrhagic complications. In this study, the safety and practical applicability of this treatment will be investigated.

Methods and analysis

A single-arm phase-II trial will be performed in 20 patients with acute peripheral arterial occlusions eligible for thrombolytic treatment. Low-dose catheter-directed thrombolysis with urokinase will be used. The investigated treatment will be performed during the first hour of thrombolysis,consisting of intravenous infusion of 4 SonoVue vials (5 mL each, 20 mL total) of microbubbles with the use of local ultrasound at the site of occlusion. Primary endpoints are the incidence of complications and technical feasibility. Secondary endpoints are angiographic and clinical success, duration of thrombolytic infusion, treatment-related mortality, amputations, additional interventions, and quality of life.

Ethics and dissemination

Ethical approval for this study was obtained in 2015 from the Medical Ethics Committee (METC) of the VU University Medical Center, Amsterdam, the Netherlands. A statement of consent for this study was given by the Dutch national competent authority. Data will be presented at national and international conferences and published in a peer-reviewed journal.

Registration: Dutch National Trial Registry: NTR4731; European Clinical Trials Database (EudraCT) of the European Medicines Agency: 2014-003469-10.

For peer review only

STRENGTHS AND LIMITATIONS

- This will be a first in man study to examine the safety and technical feasibility of therapeutic microbubbles, combined with the application of ultrasound and catheter-directed thrombolysis in peripheral arterial occlusions.
- This is a 'single arm' trial. The data will be used to inform a future large multicentre randomised controlled trial comparing conventional catheter-directed thrombolysis with microbubble and ultrasound accelerated thrombolysis
- **The present study is a non-randomized Phase II-trial, therefore the results cannot confirm benefit of sonothrombolysis for peripheral arterial occlusions, only safety and feasibility is analyzed.**
- **The present study does not compare other thrombolysis techniques or protocols.**

For peer review only

INTRODUCTION

Acute limb ischemia can be caused by a thrombus occluding an artery in an arm or leg. This is an emergency situation that can result in amputation or death if not treated successfully.[1] Intra-arterial infusion of thrombolytic agents, i.e. catheter-directed thrombolysis, can restore blood flow by dissolving the clot, as a less invasive alternative to surgical thromboembolectomy.[2] In comparison with the lysis of small arterial occlusions in patients with myocardial infarction, larger peripheral arterial occlusions require higher doses of lytic agents and infusion over a longer period of time. Inevitably, such treatment is accompanied by a risk of major hemorrhagic complications, such as hemorrhagic stroke, in up to 8% of patients.[3] Furthermore, this technique is time-consuming (several days of bed rest is usually required) and repeated angiography for treatment monitoring is needed, putting patients at risk for contrast-induced nephropathy. As a result, this leads to high morbidity rates and a significant patient burden. Methods to improve this therapy are therefore highly sought after.

A potential accelerator of thrombolytic therapy is contrast-enhanced ultrasound. Contrast agents, consisting of 5-10 µm gas-filled particles (microbubbles), have initially been used as diagnostic ultrasound contrast-enhancers. A new field of research investigates these agents for potential therapeutic purposes such as targeted drug delivery and thrombolysis.[4] The proposed mechanism of action in thrombolysis is that microbubbles can oscillate under the influence of ultrasound. At high intensities, this oscillation can lead to microbubble collapse and the production of mechanical forces on the clot surface, making the thrombus more susceptible to thrombolytics, thus accelerating thrombolysis.[5]

In early stages of clinical research, this technique has been shown to be efficient as treatment for acute cerebral stroke and acute myocardial.[6,7] Although the safety of their clinical administration in treating smaller arteries in the heart has been a topic of discussion in the past, post-marketing data for diagnostic indications showed continued safety after extensive research in more recent years.[8,9,10] For therapeutic thrombolytic purposes, this technique has been shown to be effective and safe in a porcine model of large peripheral arterial occlusions.[11] In this study, we will investigate the therapeutic application of microbubbles with ultrasound in combination with catheter-directed thrombolysis for patients with peripheral arterial occlusions. An illustrative video regarding our research project is available at https://vimeo.com/159929945/4aaf6770cf.

For peer review only

METHODS AND ANALYSIS

Study objectives

To investigate the safety and practical applicability of the therapeutic application of microbubbles and ultrasound in combination with catheter-directed thrombolysis for patients with peripheral arterial occlusions.

Design

The Microbubbles and UltraSound accelerated Thrombolysis (MUST) trial is a single-arm phase-II trial.

Primary study parameters

Main endpoints will be the safety and technical feasibility of the experimental treatment. Safety will be determined by treatment-related mortality, the occurrence of Adverse Events (AEs), Serious Adverse Events (SAEs) and Suspected Unexpected Serious Adverse Reactions (SUSARs). AEs will be defined as any undesirable experience occurring to a subject during the experimental treatment period, whether or not considered to be related to the investigational drug or intervention. SAEs will be defined as any untoward medical occurrence or effect that at any dose results in death; is life threatening (at the time of the event); requires hospitalization or prolongation of existing in-patients' hospitalization; results in persistent or significant disability or incapacity; is a new event of the trial likely to affect the safety of the subjects, such as an unexpected outcome of an adverse reaction. SUSARs, which are related to the microbubble infusion and ultrasound application, are the formation of micro embolisms resulting in occlusion of the microcirculation, hemorrhages, hypotension, heart rhythm disorders and anaphylaxis. See the paragraph *adverse events* for detailed handling procedures of AEs, SAEs and SUSARs. Hemorrhagic complications related to thrombolytic therapy will be reported according the Standardized Bleeding Definitions for Cardiovascular Clinical Trials proposed by Mehran et al.[12]

Technical feasibility will be defined as accomplishment of the experimental protocol during the first hour of thrombolysis.

Secondary study parameters

Angiographic success will be defined as dissolution of >95% of the thrombus with outflow to at least 1 crural artery. Clinical change/success will be reported according to Rutherford's recommended scale for gauging changes in clinical status (Table I). Amputations will be defined as either major (above or below knee amputation) or minor (metatarsal or toe amputation). Additional interventions will be categorized as either surgical (for example thromboembolectomy, bypass graft surgery) or percutaneous (Percutaneous Transluminal Angioplasty, stent placement) and as either required for restoration of patency or necessary for correction of underlying lesions. We will also determine microcirculation of the limb (by Laser Doppler measurements, Perimed Instruments, Järfälla, Sweden), 30-day mortality, conversion to surgery, serum fibrinogen concentrations measured during thrombolytic treatment on a daily basis, pain by Visual Analogue Scale (VAS) and quality of life by SF-36 questionnaires. The duration of thrombolysis will be defined by the time-span between initiation and completion angiography.

Table I: *Rutherford's recommended scale for gauging changes in clinical status*

Adapted from: Rutherford RB, Baker JD, Ernst C, Johnston KW, Porter JM, Ahn S, et al. Recommended standards for reports dealing with lower extremity ischemia: revised version. J Vasc Surg. 1997;26(3):517–38.

+3	Markedly improved: No ischemic symptoms, and any foot lesions completely healed; ABI essentially "normalized" (increased to more than 0.90)
+2	Moderately improved: No open foot lesions; still symptomatic but only with exercise and improved by at least one clinical chronic ischemia category; ABI not normalized but increased by more than 0.10
+1	Minimally improved: Greater than 0.10 increase in ABI* but no categorical improvement or vice versa (i.e., upward categorical shift without an increase in ABI of more than 0.10)
0	No change: No categorical shift and less than 0.10 change in ABI

For peer review only - http://bmjopen.bmj.com/site/about/guidelines.xhtml

−1	Mildly worse: No categorical shift but ABI decreased more than 0.10, or downward categorical shift with ABI decrease less than 0.10
−2	Moderately worse: One category worse or unexpected minor amputation
−3	Markedly worse: More than one category worse or unexpected major amputations
*In cases where the ABI cannot be accurately measured, an index based on the toe pressure, or any measurable pressure distal to the site of revascularization, may be substituted.	

Abbreviations: *ABI=Ankle Brachial Index*

Patients and eligibility criteria

In this phase-II trial, a total of 20 patients with acute lower limb ischemia proven by angiography will be included in our university hospital in Amsterdam, the Netherlands. Eligibility criteria are listed in Table II. Inclusion of 20 eligible patient is expected within 1.5 years. Written informed consent will be acquired by a member of the Research Team after information about the study has been provided by the treating doctor.

Table II: *Eligibility criteria*

Inclusion criteria	• Men and women older than 18 and younger than 85 years old • Patients with a maximum of 2 weeks of symptoms for lower limb ischemia due to thrombosed/occluded iliofemoral, femoropopliteal or femorocrural native arteries or iliofemoral, femoropopliteal or femorocrural venous or prosthetic bypass grafts • Patients appropriate for thrombolysis i.e. with acute lower limb ischemia class I and IIa according to the Rutherford classification<br>• Patients who understand the nature of the procedure and provide written informed consent before enrollment in the study
Exclusion criteria	• Patients with clinical complaints of acute lower limb ischemia due to thrombosis of iliofemoral, femoropopliteal or femorocrural native arteries, or iliofemoral, femoropopliteal or femorocrural venous or prosthetic bypass grafts for more than 2 weeks<br>• Patients with thrombosed popliteal aneurysms<br>• Patients with absolute contraindications for administration of antiplatelet therapy, anticoagulants or thrombolytics<br>• History of recent (less than 6 weeks) ischemic stroke, cerebral hemorrhagic or myocardial infarction • Patients with recent (less than 6 weeks) surgery<br>• Severe hypertension (diastolic blood pressure greater than 110 mm Hg, systolic blood pressure higher than 200 mm Hg) • Current malignancy or severe co-morbid condition with a life expectancy of less than 6 months<br>• Patients with uncorrected bleeding disorders (gastrointestinal ulcer, menorrhagia, liver failure)<br>• Women with childbearing potential not taking adequate contraceptives or currently breastfeeding • Pregnancy • Patients who are currently participating in another investigational drug or device study • Patients younger than 18 years or older than 85 years • Patients with contraindications for Sonovue microbubbles i.e.:<br>○ Hypersensitivity to sulphur hexafluoride or to any of the components of SonoVue ○ Recent acute coronary syndrome or clinically unstable ischemic

For peer review only

		cardiacdisease, including: evolving or ongoing myocardial infarction, unstable angina at rest within the last 7 days, significant worsening of cardiac symptoms within the last 7 days, recent coronary artery intervention or other factors suggesting clinical instability (for example, recent deterioration of ECG, laboratory or clinical findings), acute cardiac failure, Class III/IV cardiac failure, or severe rhythm disorders<br>○ Patients known to have right-to-left cardiac shunts, severe pulmonary hypertension (pulmonary artery pressure >90 mmHg), uncontrolled systemic hypertension, or adult respiratory distress syndrome<br>○ Patients with cardiovascular instability where dobutamine is contraindicated

Data handling

We will keep an electronic log of patients who fulfill the eligibility criteria, patients who are invited to participate in the study, patients recruited and patients who withdraw from the study. Reasons for non-recruitment will also be recorded. We will attempt to collect reasons for non-participation from patients who decline to take part. During the course of the study, we will document reasons for withdrawal from the study and loss to follow-up. Data will be stored electronically in Case Report Forms software with audit trail functionality and will be audited by the institutional Clinical Research Bureau (CRB). Only anonymized information will be stored and participants will only be identifiable by their unique study number, which will be kept in a separate file. Data will be securely stored on these servers for 15 years according to national guidelines.

Study procedures

Intervention

A flow chart of the patient work-up after presentation in our hospital is presented in Figure 1. Low-dose thrombolytic treatment with urokinase will be initiated following our standard institutional protocol: a catheter is placed intra-arterially in the affected artery and a bolus injection of 500,000 International Units (IU) of urokinase (Medacinase Urokinase, Medac GmbH, Hamburg, Germany) will be followed by the continuous infusion of 50,000 units of UK per hour and 9,600 IU of heparin per 24 hours. The experimental treatment consists of (in addition to the standard thrombolytic therapy) the use of local 1.8 Mhz transdermal ultrasound (Philips iE33 Ultrasound Machine, Eindhoven, the Netherlands), and the intravenous infusion of 4 vials (5 mL each, total 20 mL) of microbubbles (SonoVue, Bracco Imaging Europe B.V., the Netherlands) during the first hour of thrombolysis with urokinase. An ACIST VueJect (Bracco Imaging Europe B.V., the Netherlands) infusion pump will be used to infuse the 4 vials continuously. Ultrasound will be intermittently activated (3 seconds manual flash to burst microbubbles with Mechanical Index (MI) 1.08 (pulse duration 20 microseconds, frequency 1.8 Mhz, framerate 39 Hz), 7 seconds of visualization of inflow of the microbubbles at MI±0.11, at the site of occlusion during the first hour of thrombolysis. Criteria for discontinuation of the experimental treatment during the first hour will be the occurrence of any adverse events potentially related to the experimental treatment such as bleeding and allergic reactions.

For peer review only

For peer review only - http://bmjopen.bmj.com/site/about/guidelines.xhtml

Figure 1: *Flow chart of patient work-up after presentation*

Abbreviations: *ECG = Electro Cardiography, UK = urokinase, US = UltraSound, ABI = Ankle Brachial Index, VAS = Visual Analogue Scale, PCS= Catastrophizing Scale, QoL=Quality of Life.*

Assessments

Diagnostic measurements

Additional diagnostic measurements during admission including ECG, duplex ultrasound, angiography and microcirculation measurements (by Laser Doppler flowmetry) will be performed as depicted in Figure 1.

A duplex ultrasound will be performed every 6±2 hours to monitor for signs of revascularization. When resumption of flow is visualized by duplex ultrasound, angiography will be performed to confirm flow. Angiography will be performed at least once daily as standard procedure. Outside of routine hospital working hours, angiography will only be performed in emergencies as per standard care.

A standardized pain score (Visual Analogue Scale, 1-10), and Pain Catastrophizing Scale will be recorded every 3 hours by a nurse practitioner, research fellow or surgical resident to assess pain.

Fibrinogen monitoring

Following our standard institutional thrombolysis protocol, fibrinogen concentration will be checked during thrombolysis with the following criteria for treatment modification: If <1.0 g/L, the urokinase infusion rate will be lowered to 25,000 IU/h; if <0.5 g/L, thrombolysis must be aborted temporarily and replaced by normal saline infusion. Three hours following treatment modifications, fibrinogen concentration will be reevaluated and when >1.0 g/L thrombolysis will be restarted at an initial low dose urokinase of 50,000 IU/h.

Post-procedural anticoagulation

After successful thrombolysis, the patient will be heparinized with low-molecular weight heparin (fraxiparine) dosed based on body weight: <50 kg: 2 times a day 3,800 IU (= 0.4 mL), 50-80 kg: 2 times a day 5,700 IU (= 0.6 mL), >80 kg: 2 times a day 7,600 IU (= 0.8 mL).

Concomitant therapy with coumarin derivatives will also be started at that time. Activated partial thromboplastin time (aPTT) will be measured daily during heparin treatment. The target range international normalized ratio (INR) will be 2.5 to 3.5; if this value is reached, heparinization will be stopped and coumarin treatment will be continued.

Follow-up

Outpatient follow-up will take place at specific time points for a total duration of 1 year, measurements performed during follow-up visits are depicted in Figure 1.

Adverse events

Adverse events will be recorded in detail in the electronic patient record. Any serious adverse events that occur after joining the trial will be reported to the accredited Medical Ethics Committee of our institution according to national and institutional guidelines. All adverse events will be followed up until they have abated, or until a stable situation has been reached. Depending on the event, follow up may require additional tests or medical procedures as indicated, and/or referral to a general physician or medical specialist. An interim analysis after 10 patients will be performed and if serious adverse events have occurred, we will discuss the continuation of the study. The study will be prematurely terminated if 2 or more intracranial bleedings occur or more than 5 allergic reactions.

Statistical analysis

For peer review only

Categorized epidemiologic/descriptive patient variables are summarized with frequencies and will be analyzed with Fischer's exact test or the Pearson Chi-squared test. To avoid possible violations of the assumptions for parametric testing, such as a normal distribution pattern, we will employ non-parametric methods such as a Spearman rank correlation and a Mann-Whitney U test in the case of a skewed distribution or log transformation. For associations of two outcome measurements, we will use a correlation (Spearman rank) or single regression analysis. We will analyze the following outcomes by means of Kaplan-Meier curves: patency rate, amputation-free rate, and intervention-free rate. We will assess heterogeneity in prognostic factors as a secondary analysis by means of Chi-squared tests. All tests will be performed two-sided, and a $p<0.05$ will be considered to be statistically significant.

ETHICS AND DISSEMINATION

The study will be conducted according to the principles of the Declaration of Helsinki (Brazil, October 2013), and in accordance with the Medical Research Involving Human Subjects Act (WMO). An Investigator Site File will be produced in advance of the study conforming to institutional guidelines. Furthermore we will create Case Report Forms by using GCP and 21 CFR Part 11 compliant software, to handle patient data.

The study has been registered in the Dutch Trial Register, at the Dutch National Central Committee on Research Involving Human Subjects (CCMO) and in the European Clinical Trials Database (EudraCT) of the European Medicines Agency. Any protocol amendments during the study will be communicated and changed accordingly in the relevant registries after approval of the institutional Medical Ethics Committee. The results of this study will be submitted for publication in a peer-reviewed journal, regardless of the outcome of this study, according to the CCMO statement on publication policy. Data will also be presented at international conferences.

For peer review only

DISCUSSION

The Microbubbles and UltraSound-accelerated Thrombolysis (MUST) trial is a phase-II single-arm clinical trial. In this study the safety and feasibility of an experimental ultrasound technique will be investigated for the first time in patients with large peripheral arterial occlusions.

We believe that this procedure is safe and can accelerate thrombolysis, thereby allowing for reduction of thrombolytic dosage, which in turn reduces the risk of major hemorrhagic complications.

An experimental bolus therapy with microbubbles and ultrasound could accelerate thrombolysis because at high ultrasound intensities microbubbles can collapse, resulting in mechanical forces on the clot surface. The formation of small channels in the thrombus lead to exposure of a larger total surface susceptible to thrombolytics.[5]

In regard to the therapeutic application of contrast agents, several studies have been performed in patients with ischemic stroke and myocardial infarction. A systematic review of sonothrombolysis shows that this treatment option improves clinical and long-term outcomes, while potentially reducing bleeding risk, in patients with ischemic stroke.[13] Nevertheless, dose escalation studies show that the safety (in terms of bleeding and micro emboli) needs to be further investigated before enrolling patients in phase-III trials.[14] Few and heterogeneous studies examined the therapeutic application of sonothrombolysis in patients with myocardial infarction. Although pilot studies affirm safety and feasibility, the application of therapeutic ultrasound with longer pulse durations (20 microseconds vs. 5 microseconds) was reported to result in unexpected coronary vasoconstriction in a recent clinical trial.[15]
Potential reported mechanisms for this effect are the summative effect of myocardial ischemia, reperfusion damage and long-pulse-duration sonoporation on endothelial damage, all leading to calcium overload. However, patients with peripheral arterial occlusions are mostly chronic vascular patients who often have received previous treatments in the respective artery, for example thrombolytic therapy, percutaneous transluminal angioplasty, thromboembolectomy or bypass surgery. The mechanical manipulation of the vascular wall during all these treatments is extensive. Furthermore, during standard thrombolytic treatment, arteries are manipulated and perforated on purpose to insert guide wires and catheters. Hence, vascular spasms during these peripheral treatments are normal and non-threatening to the patient, in contrast to spasms in small coronary arteries.

The administration of ultrasound contrast agents has been accompanied by important discussions regarding safety concerns in the past[8,16]. As a response to the occurrence of SAEs, the US Food and Drug Administration (FDA) issued a labeling change and warnings for contrast agents in 2007. Consequently, new studies on the risks of contrast agents were performed and these established their safety.[17] In Europe the European Medicines Agency conducted an extensive evaluation of the contrast agent SonoVue and concluded with the decision to remove its previously instated contraindications.[18]

In regard to SonoVue contrast agent dose regimens, the maximum approved dose for diagnostic indications is 4.8 mL injected over a short time-period.[19] For therapeutic purposes, we anticipate a higher total dose of 20 mL in one hour. In an 80 kg patient this equates to 0.25 mL/kg. Clinical studies have reported cumulative doses up to 0.6 mL/kg of SonoVue contrast agent without the occurrence of adverse events or side effects.[20] During the experimental protocol with microbubble infusion, patients will be continuously monitored.

For peer review only - http://bmjopen.bmj.com/site/about/guidelines.xhtml

As with all contrast agents, the risk of anaphylactic reactions to contrast remains. Therefore, administration of contrast agents in a center with full resuscitation possibilities is mandatory. Furthermore, during the first hours of administration, monitoring of vital parameters of patients is important.

In this study, thrombolysis is performed with the fibrinolytic urokinase, which is the most used fibrinolytic agent for the treatment of peripheral arterial occlusions worldwide and is standard care in the Netherlands. Some countries use tissue plasminogen activator for this indication. A Cochrane review on the topic states that there is no evidence that (r)t-PA is more effective than urokinase for patients with peripheral arterial occlusion.[21]

If the application of microbubbles and ultrasound concomitant to catheter-directed thrombolysis is shown to be safe and technically feasible based on this phase-II trial, we anticipate a funding application for a larger randomized controlled trial with a comparative group to assess and compare efficacy of this treatment.

Although the efficacy of the currently described protocol cannot be adequately compared within this study design, we will discuss the outcomes relative to a historic control group that had previously received our standard hospital thrombolysis protocol.[22]

Successful thrombolysis is strongly predictive of amputation-free survival with vascular patency for at least one year.[23] A longer duration of thrombolysis inevitably exposes a patient to a higher thrombolytic dose and higher risk of hemorrhage, in addition to an already increased patient burden because of prolonged bed rest. Therefore ultimately, acceleration of thrombolysis with microbubbles could benefit the patient because of a shorter therapy time, a lower risk of hemorrhagic complications and a decrease in patient burden.

ACKNOWLEDGEMENTS:
We would like to thank Laura van Wieringen, Lloyd Belliot, Joyce Lu, Jorn Meekel, Ted van Schaik and Sean Matheiken for their assistance in preparation of this study.

AUTHOR CONTRIBUTIONS

HE, JN, WW and KK planned and designed the research, HE wrote the manuscript, HE, JN, RL and KK critically revised the manuscript and all authors and MUST collaborators approved the final version of the manuscript.

FUNDING: This study is supported by the Departments of Surgery, Radiology, Cardiology and Physiology of the VU University Medical Center Amsterdam, the Netherlands and by a unrestricted research grant from LamePro B.V., the Netherlands. LamePro B.V. has no role in the study design, collection, management, analysis, and interpretation of data, nor in writing of the report, submitting the report for publication or ultimate authority on any of the previous mentioned.

COMPETING INTERESTS: none

For peer review only

REFERENCES

1. Creager, M. A., Kaufman, J. A., & Conte, M. S. Clinical practice. Acute limb ischemia. *N Engl J Med*, 2012. 366(23), 2198–2206.
2. Berridge, D. C., Kessel, D., & Robertson, I. Surgery versus thrombolysis for acute limb ischaemia: initial management. *Cochrane Database Syst Rev*, (3) (2013), CD002784.
3. van den Berg, J. C. Thrombolysis for acute arterial occlusion. *J Vasc Surg*, 2009. 52(2), 512–515.
4. Dijkmans, P. A., Juffermans, L. J. M., Musters, R. J. P., Wamel, A. Van, Cate, F. J., Gilst, W. Van, Kamp, O. Microbubbles and ultrasound: from diagnosis to therapy. *Eur J Echocardiogr.* 2004 Aug;5(4):245-56.
5. Everbach, E. C., & Francis, C. W. (2000). Cavitational mechanisms in ultrasound-accelerated thrombolysis at 1 MHz. *Ultrasound Med Biol*, 2000 26(7), 1153–1160.
6. Balucani C, Alexandrov A V. Ultrasound- and microspheres-enhanced thrombolysis for stroke treatment: state of the art. *Curr Cardiol Rep*. 2010;12(1):34–41.
7. Slikkerveer J, Kleijn SA, Appelman Y, Porter TR, Veen G, van Rossum AC, et al. Ultrasound enhanced prehospital thrombolysis using microbubbles infusion in patients with acute ST elevation myocardial infarction: pilot of the Sonolysis study. *Ultrasound Med Biol*. 2012;38(2):247–52.
8. Dijkmans PA, Visser CA, Kamp O. Adverse reactions to ultrasound contrast agents: Is the risk worth the benefit? *Eur J Echocardiogr*. 2005 *Oct;6(5):363-6.*
9. Roos ST, Juffermans LJM, van Royen N, van Rossum AC, Xie F, Appelman Y, et al. Unexpected High Incidence of Coronary Vasoconstriction in the Reduction of Microvascular Injury Using Sonolysis (ROMIUS) Trial. *Ultrasound Med Biol*. 2016 May 5 (Epub ahead of print).
10. Appis a. W, Tracy MJ, Feinstein SB. Update on the safety and efficacy of commercial ultrasound contrast agents in cardiac applications. Echo Res Pract. 2015;2(2):R55–62.
11. Ebben HP, Nederhoed JH, Slikkerveer J, Kamp O, Tangelder GWJM, Musters RJP, et al. Therapeutic application of contrast-enhanced ultrasound and low-dose urokinase for thrombolysis in a porcine model of acute peripheral arterial occlusion. *J Vasc Surg*. 2015 Aug;62(2):477–85.
12. Mehran R, Rao S V., Bhatt DL, Gibson CM, Caixeta A, Eikelboom J, et al. Standardized Bleeding Definitions for Cardiovascular Clinical Trials: A Consensus Report From the Bleeding Academic Research Consortium. *Circulation*. 2011 Jun 13;123(23):2736–47.
13. Roos ST, Juffermans LJM, Slikkerveer J, Unger EC, Porter TR, Kamp O. Sonothrombolysis in acute stroke and myocardial infarction: A systematic review. *IJC Hear Vessel*.; 2014;4:1–6.
14. Molina CA, Barreto AD, Tsivgoulis G, Sierzenski P, Malkoff MD, Rubiera M, et al. Transcranial ultrasound in clinical sonothrombolysis (TUCSON) trial. *Ann Neurol*.; 2009;66(1):28–38.
15. Roos ST, Juffermans LJM, van Royen N, van Rossum AC, Xie F, Appelman Y, et al. Unexpected High Incidence of Coronary Vasoconstriction in the Reduction of Microvascular Injury Using Sonolysis (ROMIUS) Trial. *Ultrasound Med Biol* [Internet]. 2016;1–10.
16. Douglas PS, Weyman AE, Lindner JR, Wei K.Contrast echocardiography: past, present, and...future? *JACC Cardiovasc Imaging.* 2008 Jan;1(1):107-10.
17. FDA Advisory Committee Briefing Document Cardiovascular and Renal Drugs Advisory Committee June 24th 2008. (Accessed on 23-05-2016, available at http://www.fda.gov/ohrms/dockets/ac/08/briefing/2008- 4369b1-01.pdf).
18. Sonovue: EPAR-Assessment Report-Variation. (Accessed on 23-05-2016, available at http://www.ema.europa.eu/docs/en_GB/document_library/EPAR_-_Assessment_Report_-_Variation/human/000303/WC500170218.pdf)

For peer review only - http://bmjopen.bmj.com/site/about/guidelines.xhtml

19. EMA Sonovue Summary of Product Characteristics (SmPC) (Accessed on 23-05-2016, available at http://www.ema.europa.eu/docs/en_GB/document_library/EPAR_-_Product_Information/human/000303/WC500055380.pdf)
20. Bokor D, Chambers JB, Rees PJ, Mant TG, Luzzani F, Spinazzi A. Clinical safety of SonoVue, a new contrast agent for ultrasound imaging, in healthy volunteers and in patients with chronic obstructive pulmonary disease. *Invest Radiol*.; 2001;36(2):104–9.
21. Robertson I, Kessel DO, Berridge DC. Fibrinolytic agents for peripheral arterial occlusion. *Cochrane Database Syst Rev*. 2013 Dec 19;12:CD001099.
22. Ebben HP, Nederhoed JH, Lely RJ, Meijerink MR, van der Meijs BB, Wisselink W, et al. Low-dose Thrombolysis for Thromboembolic Lower Extremity Arterial Occlusions is Effective Without Major Hemorrhagic Complications. *Eur J Vasc Endovasc Surg*. 2014 Nov;48(5):551-8.
23. G. Plate, S. Oredsson, J. Lanke. When is thrombolysis for acute lower limb ischemia worthwhile? *Eur J Vasc Endovasc Surg*, 37 (2) (2009), pp. 206–212

For peer review only

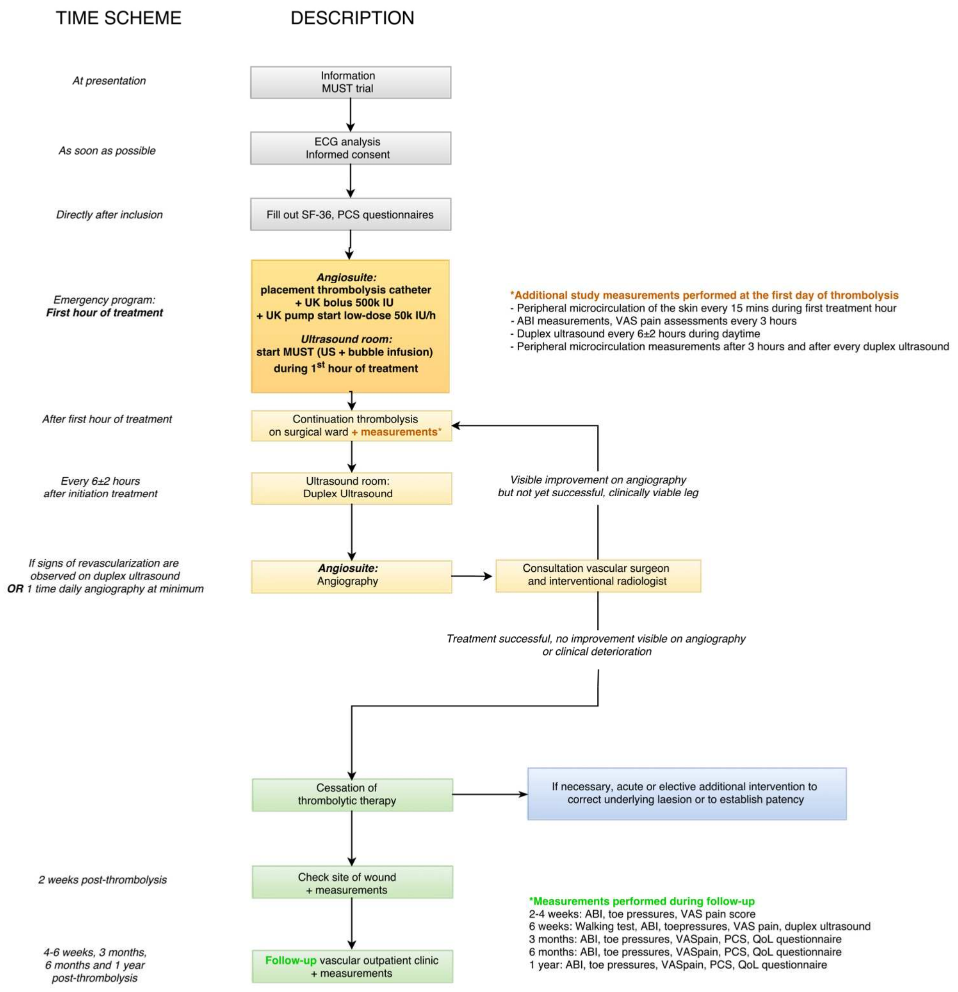

Flow chart of patient work-up after presentation.
Abbreviations: ECG = Electro Cardiography, UK = urokinase, US = UltraSound, ABI = Ankle Brachial Index, VAS = Visual Analogue Scale, PCS= Catastrophizing Scale, QoL=Quality of Life.

Figure 1
157x167mm (300 x 300 DPI)

Korte proefpersoneninformatiebrief

MUST Trial

Microbubbels en ultrageluid toegevoegd aan een stolseloplossend medicijn

Volledige Engelse titel:
The application of contrast enhanced ultrasound (CEUS) to facilitate thrombolysis in patients with acute peripheral arterial occlusions.

VU medisch centrum

For peer review only - http://bmjopen.bmj.com/site/about/guidelines.xhtml

Geachte heer/mevrouw,

Wij vragen u vriendelijk om mee te doen aan een medisch-wetenschappelijk onderzoek waarin een nieuwe behandeling voor een vaatafsluiting in het been wordt onderzocht. U beslist zelf of u wilt meedoen.

In deze korte informatiebrief wordt u uitgelegd wat het onderzoek inhoudt. Na afloop van de behandeling ontvangt u een uitgebreide informatiebrief over het onderzoek.

Wat wordt er onderzocht en waarom?

In dit onderzoek wordt de behandeling met echo en contrast bovenop de standaard stolseloplossende behandeling onderzocht bij patiënten met een stolsel in hun beenvaten. Het doel is de trombolysebehandeling te versnellen met behulp van echogeluidsgolven en microbubbles. Microbubbles zijn kleine gasbelletjes die normaal als contrast bij echo worden gebruikt. Ze werden dus al vaker in patiënten gebruikt om de echobeelden te verduidelijken. Wij gebruiken de trilkrachten van de microbubbles. Zij kunnen namelijk trillen door de echogeluidsgolven en het stolsel in uw vat mogelijk sneller oplossen.

Het toedienen van contrast bij echo wordt al jaren veilig bij patiënten toegepast om betere beeldvorming te krijgen en dit contrast is niet belastend voor de nieren. De inzet van echo met contrast bij het oplossen van een stolsel wordt ook in patiënten met hart- en herseninfarcten al veel onderzocht.

Wat staat u te wachten als u deelneemt aan dit onderzoek?

Nadat u schriftelijk toestemming heeft gegeven om deel te nemen aan het onderzoek zal er een korte vragenlijst worden afgenomen (duur ongeveer 2 minuten) en zal de standaard stolseloplossende behandeling gestart worden. Informatie over de stolseloplossende behandeling wordt gegeven door de arts van de afdeling of de behandelend chirurg.

For peer review only

For peer review only - http://bmjopen.bmj.com/site/about/guidelines.xhtml

De experimentele behandeling bestaat uit de toevoeging van echo met contrast gedurende het eerste uur van de standaard behandeling. Hiervoor wordt via een infuus in uw arm gedurende een uur contrast toegediend. Gedurende 1 uur zal dit infuus inlopen en zal een radioloog of echolaborant tegelijkertijd op uw been echogolven afgeven. De rest van de behandeling is zoals de standaard stolseloplossende behandeling. Gedurende de rest van de opname en bij een aantal controlebezoeken op de poli-kliniek zullen extra metingen worden verricht zoals
metingen van de doorbloeding van de huid, bloeddruk metingen in arm en been en het afnemen van een pijnscore en korte vragenlijst.
In de volgende afbeelding staat schematisch weergegeven hoe het gehele behandelingstraject eruit ziet voor u. **Dikgedrukt zijn alle handelingen en metingen die extra zijn door deel te nemen aan dit onderzoek** ten opzichte van een normale

For peer review only

For peer review only - http://bmjopen.bmj.com/site/about/guidelines.xhtml

trombolysebehandeling.

Tijdsmoment	*Omschrijving*
Direct bij opname	**Informatie MUST trial**
Zo snel mogelijk	**Analyse hartfilmpje Toestemmingsverklaring**
Direct na besluit tot deelname aan onderzoek	**Invullen vragenlijst**
Via spoed programma: Eerste uur van de behandeling	- Radiologieruimte: plaatsing slangetje in beenvat voor trombolyse + start stolseloplossende medicatie<br>**- Echokamer: start MUST (echo + contrastmiddel)**
Na 1e uur van behandeling	Vervolg trombolyse behandeling op opnameafdeling
Elke 6 uur na start behandeling	**Echokamer: Echo onderzoek been**
Als tekenen van verbetering worden gezien met echo **OF** minimaal 1x per dag	CTA scan
Gebaseerd op resultaten CTA scan na overleg vaatchirurg en interventieradioloog.	Stoppen trombolyse behandeling
2 weken na ontslag	Wondcontrole op polikliniek en **metingen**
**4 weken,** 6 weken, 3 maanden, 6 maanden en 1 jaar na ontslag	Controle op polikliniek en **metingen**

Toelichting enkele termen:

Een **CTA scan** is een speciale CT scan waarbij met contrastmiddel naar de vaten gekeken kan worden. De A staat voor angiografie. Deze scan is onderdeel van de standaard trombolyse behandeling en hiermee wordt gekeken naar de doorgankelijkheid van de beenvaten. Het contrastmiddel dat hierbij gebruikt wordt is een ander

For peer review only

For peer review only - http://bmjopen.bmj.com/site/about/guidelines.xhtml

contrastmiddel dan gebruikt bij de echo tijdens het eerste uur van de behandeling waar onderzoek naar gedaan wordt. Het contrast gebruikt tijdens de CTA scan is mogelijk schadelijk voor de nieren. Afhankelijk van uw nierfunctie worden hier mogelijk speciale voorbereidingen voor gedaan zoals het geven van vocht voor en na de scan om dit te voorkomen.

De controles op de polikliniek worden ook gedaan bij de standaard trombolysebehandeling, na 4 weken is er alleen een extra controlemoment bij de patiënten die deelnemen aan het onderzoek. De metingen die bij u gedaan worden tijdens dit controlebezoek zijn:

- Meting bloeddruk (armen, enkels, tenen)
- Pijnscore

Tijdens de controle op de polikliniek na 6 weken zal er behalve bovenstaande metingen ook een looptest en een echo gemaakt worden.

Na 3 en 6 maanden en na 1 jaar zal er ook een vragenlijst op de iPad ingevuld moeten worden met vragen over pijn en de kwaliteit van leven.

Wat zijn mogelijke voor- en nadelen van deelname aan dit onderzoek?

Mogelijk heeft u voordeel van de behandeling omdat deze door de nieuwe techniek verkort wordt. Mogelijk is hierdoor ook het risico op bloedingscomplicaties gedurende de behandeling verminderd. Tevens kan het onderzoek voor de toekomst nuttige gegevens opleveren voor de behandeling.

Nadelen van deelname zijn dat er gedurende het eerste uur van behandeling meer handelingen worden verricht en dat er gedurende de opname meer metingen worden verricht bij u. Tevens moet u voor en na behandeling bij terugkomst op de poli enkele malen een korte vragenlijst invullen. De mogelijke bijwerkingen van het echo contrast middel zijn in het algemeen niet ernstig en van voorbijgaande aard. De meest gemelde bijwerkingen zijn hoofdpijn (2%), pijn op de plaats van injectie (1%), reacties op de plaats van injectie waaronder bloeduitstortingen, warmte, verminderde gevoelswaarneming (2%). In enkele gevallen is een allergische reactie, verandering op het hartfilmpje (hier merkt u zelf meestal niets van), bloeddruk en in sommige

For peer review only

For peer review only - http://bmjopen.bmj.com/site/about/guidelines.xhtml

laboratoriumwaarden waargenomen (tijdelijke toename van witte bloedcellen, hier merkt u zelf meestal niets van).

Mocht u vragen hebben over het onderzoek of de behandeling dan kunt u terecht bij uw behandelend arts of een van de onderzoekers.

Met vriendelijke groet,

J.H. Nederhoed (vaatchirurg en onderzoeker), K.K. Yeung (chirurg in opleiding en coördinerend onderzoeker), H.P. Ebben (onderzoeker)

Mede namens,

Vaatchirurgen: H.M.E. Coveliers, A.W.J. Hoksbergen, J.A. Rauwerda, J.D. Blankensteijn, W. Wisselink
Interventieradioloog: R.J. Lely

Toestemmingsformulier
(Voor volwassenen die zelfstandig beslissingen kunnen nemen (wilsbekwaam zijn).)

Microbubbles en ultrageluid bij trombolyse

For peer review only

For peer review only - http://bmjopen.bmj.com/site/about/guidelines.xhtml

NL46636.029.14 18-11-2014

Ik heb de informatiebrief voor de proefpersoon gelezen. Ik kon aanvullende vragen stellen. Mijn vragen zijn genoeg beantwoord. Ik had genoeg tijd om te beslissen of ik meedoe.

Ik weet dat meedoen helemaal vrijwillig is. Ik weet dat ik op ieder moment kan beslissen om toch niet mee te doen. Daarvoor hoef ik geen reden te geven.

Ik geef toestemming om mijn huisarts te vertellen dat ik meedoe aan dit onderzoek. (indien van toepassing)

Ik geef toestemming om de specialist(en) die mij behandelt te vertellen dat ik meedoe aan dit onderzoek. (indien van toepassing)

Ik weet dat sommige mensen mijn gegevens kunnen zien. Die mensen staan vermeld in de Algemene brochure.

Ik geef toestemming om mijn gegevens te gebruiken, voor de doelen die in de informatiebrief staan.

Ik geef toestemming om mijn onderzoeksgegevens 15 jaar na afloop van dit onderzoek te bewaren.

Ik geef wel/geen* toestemming om mijn lichaamsmateriaal 15 jaar na afloop van dit onderzoek te bewaren, zodat dit in de toekomst misschien kan worden gebruikt voor onderzoek zoals beschreven in de informatiebrief. (indien van toepassing).

Ik wil meedoen aan dit onderzoek.

For peer review only

For peer review only - http://bmjopen.bmj.com/site/about/guidelines.xhtml

Naam proefpersoon:

Handtekening: Datum: __ / __ / __

Ik verklaar hierbij dat ik deze proefpersoon volledig heb geïnformeerd over het genoemde onderzoek.

Als er tijdens het onderzoek informatie bekend wordt die de toestemming van de proefpersoon zou kunnen beïnvloeden, dan breng ik hem/haar daarvan tijdig op de hoogte.

Naam onderzoeker (of diens vertegenwoordiger):

Handtekening: Datum: __ / __ / __

Aanvullende informatie is gegeven door (indien van toepassing):

Naam:

Functie:

Handtekening: Datum: __ / __ / __

---

* Doorhalen wat niet van toepassing is.

For peer review only

For peer review only - http://bmjopen.bmj.com/site/about/guidelines.xhtml

STANDARD PROTOCOL ITEMS: RECOMMENDATIONS FOR INTERVENTIONAL TRIALS

SPIRIT 2013 Checklist: Recommended items to address in a clinical trial protocol and related documents*

BOLD AND ITALIC TEXT MEANS CHECKED (manuscript page number for reference)

Section/item	Item No	Description
**Administrative information**		
Title	***1***	***Descriptive title identifying the study design, population, interventions, and, if applicable, trial acronym (page 1)***
Trial registration	***2a***	***Trial identifier and registry name. If not yet registered, name of intended registry (page 2)***
	2b	***All items from the World Health Organization Trial Registration Data Set (page 2)***
Protocol version	***3***	***Date and version identifier (page 1)***
Funding	***4***	***Sources and types of financial, material, and other support (page 12)***
Roles and responsibilities	***5a***	***Names, affiliations, and roles of protocol contributors (page 12)***
	5b	***Name and contact information for the trial sponsor (page 1)***
	5c	***Role of study sponsor and funders, if any, in study design; collection, management, analysis, and interpretation of data; writing of the report; and the decision to submit the report for publication, including whether they will have ultimate authority over any of these activities (page 12)***
	5d	***Composition, roles, and responsibilities of the coordinating centre, steering committee, endpoint adjudication committee, data management team, and other individuals or groups overseeing the trial, if applicable (see Item 21a for data monitoring committee) (NOT APPLICABLE (NA))***
**Introduction**		
Background and rationale	***6a***	***Description of research question and justification for undertaking the trial, including summary of relevant studies (published and unpublished) examining benefits and harms for each intervention (page 4)***

For peer review only

	6b	*Explanation for choice of comparators → NOT APPLICABLE*
Objectives	*7*	*Specific objectives or hypotheses (page 5)*
Trial design	*8*	*Description of trial design including type of trial (eg, parallel group, crossover, factorial, single group), allocation ratio, and framework (eg, superiority, equivalence, noninferiority, exploratory) (page 5)*
**Methods: Participants, interventions, and outcomes**		
Study setting	*9*	*Description of study settings (eg, community clinic, academic hospital) and list of countries where data will be collected. Reference to where list of study sites can be obtained (page 6)*
Eligibility criteria	*10*	*Inclusion and exclusion criteria for participants. If applicable, eligibility criteria for study centres and individuals who will perform the interventions (eg, surgeons, psychotherapists) (page 6)*
Interventions	*11a*	*Interventions for each group with sufficient detail to allow replication, including how and when they will be administered (pages 7,8)*
	11b	*Criteria for discontinuing or modifying allocated interventions for a given trial participant (eg, drug dose change in response to harms, participant request, or improving/worsening disease) (page 7)*
	11c	*Strategies to improve adherence to intervention protocols, and any procedures for monitoring adherence (eg, drug tablet return, laboratory tests) (NA)*
	11d	*Relevant concomitant care and interventions that are permitted or prohibited during the trial (page 8)*
Outcomes	*12*	*Primary, secondary, and other outcomes, including the specific measurement variable (eg, systolic blood pressure), analysis metric (eg, change from baseline, final value, time to event), method of aggregation (eg, median, proportion), and time point for each outcome. Explanation of the clinical relevance of chosen efficacy and harm outcomes is strongly recommended (page 5)*
Participant timeline	*13*	*Time schedule of enrolment, interventions (including any run-ins and washouts), assessments, and visits for participants. A schematic diagram is highly recommended (see Figure) (page 8)*
Sample size	*14*	*Estimated number of participants needed to achieve study objectives and how it was determined, including clinical and statistical assumptions supporting any sample size calculations (page 6)*

For peer review only - http://bmjopen.bmj.com/site/about/guidelines.xhtml

Recruitment	*15*	*Strategies for achieving adequate participant enrolment to reach target sample size (page 6)*
Methods: Assignment of interventions (for controlled trials)		
Allocation:		
Sequence generation	*16a*	*Method of generating the allocation sequence (eg, computer-generated random numbers), and list of any factors for stratification. To reduce predictability of a random sequence, details of any planned restriction (eg, blocking) should be provided in a separate document that is unavailable to those who enrol participants or assign interventions (NA)*
Allocation concealment mechanism	*16b*	*Mechanism of implementing the allocation sequence (eg, central telephone; sequentially numbered, opaque, sealed envelopes), describing any steps to conceal the sequence until interventions are assigned (NA)*
Implementation	*16c*	*Who will generate the allocation sequence, who will enrol participants, and who will assign participants to interventions (NA)*
Blinding (masking)	*17a*	*Who will be blinded after assignment to interventions (eg, trial participants, care providers, outcome assessors, data analysts), and how (NA)*
	17b	*If blinded, circumstances under which unblinding is permissible, and procedure for revealing a participant's allocated intervention during the trial (NA)*
Methods: Data collection, management, and analysis		
Data collection methods	*18a*	*Plans for assessment and collection of outcome, baseline, and other trial data, including any related processes to promote data quality (eg, duplicate measurements, training of assessors) and a description of study instruments (eg, questionnaires, laboratory tests) along with their reliability and validity, if known. Reference to where data collection forms can be found, if not in the protocol (page 9)*
	18b	*Plans to promote participant retention and complete follow-up, including list of any outcome data to be collected for participants who discontinue or deviate from intervention protocols (pages 8,9)*
Data management	*19*	*Plans for data entry, coding, security, and storage, including any related processes to promote data quality (eg, double data entry; range checks for data values). Reference to where details of data management procedures can be found, if not in the protocol (page 7)*

Statistical methods	*20a*	*Statistical methods for analysing primary and secondary outcomes. Reference to where other details of the statistical analysis plan can be found, if not in the protocol (page 9)*
	20b	*Methods for any additional analyses (eg, subgroup and adjusted analyses) (NA)*
	20c	*Definition of analysis population relating to protocol non-adherence (eg, as randomised analysis), and any statistical methods to handle missing data (eg, multiple imputation) (page 9)*
Methods: Monitoring		
Data monitoring	*21a*	*Composition of data monitoring committee (DMC); summary of its role and reporting structure; statement of whether it is independent from the sponsor and competing interests; and reference to where further details about its charter can be found, if not in the protocol. Alternatively, an explanation of why a DMC is not needed (NA)*
	21b	*Description of any interim analyses and stopping guidelines, including who will have access to these interim results and make the final decision to terminate the trial (NA)*
Harms	22	Plans for collecting, assessing, reporting, and managing solicited and spontaneously reported adverse events and other unintended effects of trial interventions or trial conduct (page 9)
Auditing	*23*	*Frequency and procedures for auditing trial conduct, if any, and whether the process will be independent from investigators and the sponsor (page 7)*
Ethics and dissemination		
Research ethics approval	*24*	*Plans for seeking research ethics committee/institutional review board (REC/IRB) approval (page 2)*
Protocol amendments	*25*	*Plans for communicating important protocol modifications (eg, changes to eligibility criteria, outcomes, analyses) to relevant parties (eg, investigators, REC/IRBs, trial participants, trial registries, journals, regulators) (page 10)*
Consent or assent	*26a*	*Who will obtain informed consent or assent from potential trial participants or authorised surrogates, and how (see Item 32) (page 6)*
	26b	*Additional consent provisions for collection and use of participant data and biological specimens in ancillary studies, if applicable (NA)*

For peer review only

Confidentiality	***27***	***How personal information about potential and enrolled participants will be collected, shared, and maintained in order to protect confidentiality before, during, and after the trial (page 7)***
Declaration of interests	***28***	***Financial and other competing interests for principal investigators for the overall trial and each study site (page 12)***
Access to data	***29***	***Statement of who will have access to the final trial dataset, and disclosure of contractual agreements that limit such access for investigators (page 7)***
Ancillary and post-trial care	***30***	***Provisions, if any, for ancillary and post-trial care, and for compensation to those who suffer harm from trial participation (NA)***
Dissemination policy	***31a***	***Plans for investigators and sponsor to communicate trial results to participants, healthcare professionals, the public, and other relevant groups (eg, via publication, reporting in results databases, or other data sharing arrangements), including any publication restrictions (page 10)***
	31b	***Authorship eligibility guidelines and any intended use of professional writers (page 12)***
	31c	***Plans, if any, for granting public access to the full protocol, participant-level dataset, and statistical code (NA)***
**Appendices**		
Informed consent materials	***32***	***Model consent form and other related documentation given to participants and authorised surrogates (attached in appendices)***
Biological specimens	***33***	***Plans for collection, laboratory evaluation, and storage of biological specimens for genetic or molecular analysis in the current trial and for future use in ancillary studies, if applicable (NA)***

*It is strongly recommended that this checklist be read in conjunction with the SPIRIT 2013 Explanation & Elaboration for important clarification on the items. Amendments to the protocol should be tracked and dated. The SPIRIT checklist is copyrighted by the SPIRIT Group under the Creative Commons "Attribution-NonCommercial-NoDerivs 3.0 Unported" license.

For peer review only

BMJ Open

Microbubbles and UltraSound-accelerated Thrombolysis (MUST) for peripheral arterial occlusions: protocol for a phase-II single arm trial

Journal:	*BMJ Open*
Manuscript ID	bmjopen-2016-014365.R2
Article Type:	Protocol
Date Submitted by the Author:	06-Mar-2017
Complete List of Authors:	Ebben, Harm; VU University Medical Center, Surgery<br>Nederhoed, Johanna; VU University Medical Center, Surgery<br>Lely, Rutger; VU University Medical Center, Radiology<br>Wisselink, Willem; VU University Medical Center, Surgery<br>Yeung, Kakkhee; VU University Medical Center, Surgery<br>MUST, Collaborators; VU University Medical Center, Surgery, Radiology, Cardiology, Physiology
**Primary Subject Heading**:	Surgery
Secondary Subject Heading:	Cardiovascular medicine, Surgery
Keywords:	peripheral arterial occlusion, thrombolysis, microbubbles, sonothrombolysis
Note: The following files were submitted by the author for peer review, but cannot be converted to PDF. You must view these files (e.g. movies) online.	
Microbubbles in Vascular Surgery (Enhanced)-HD Ebben et al.mp4	

SCHOLARONE™
Manuscripts

Microbubbles and UltraSound-accelerated Thrombolysis (MUST) for peripheral arterial occlusions: protocol for a phase-II single arm trial

Harm P. Ebben[1,4], Johanna H. Nederhoed[1], Rutger J. Lely[2], Willem Wisselink[1], Kak Khee Yeung[1,4] & MUST collaborators*

*Arjan W.J. Hoksbergen[1], Jan D. Blankensteijn[1], Bram B. van der Meijs[2], Martijn R. Meijerink[2], Otto Kamp[3], J. Slikkerveer[3], René J.P. Musters[4], Geert-Jan Tangelder[4]

Departments of [1]Surgery, [2]Radiology, [3]Cardiology and [4]Physiology, VU University Medical Center, Amsterdam, the Netherlands

Corresponding author

Dr. K.K. Yeung, MD, PhD
VU University Medical Center
P.O. Box 7057
1007 MB Amsterdam
The Netherlands
tel +31 (0)6 14 27 87 25

For peer review only

Key words: peripheral arterial occlusion, thrombolysis, microbubbles, ultrasound, sonothrombolysis

Word count: 2626 (*Excluding title page, abstract, references, figures and tables.)*

Protocol version January 2017

Abstract

Introduction

Acute peripheral arterial occlusions can be treated with intra-arterial catheter-directed thrombolysis as an alternative to surgical thromboembolectomy. Although less invasive, this treatment is time-consuming and carries a significant risk of hemorrhagic complications. Contrast-enhanced ultrasound using microbubbles could accelerate dissolution of thrombi by thrombolytic medications due to mechanical effects caused by oscillation; this could allow for lower dosages of thrombolytics and faster thrombolysis, thereby reducing the risk of hemorrhagic complications. In this study, the safety and practical applicability of this treatment will be investigated.

Methods and analysis

A single-arm phase-II trial will be performed in 20 patients with acute peripheral arterial occlusions eligible for thrombolytic treatment. Low-dose catheter-directed thrombolysis with urokinase will be used. The investigated treatment will be performed during the first hour of thrombolysis,consisting of intravenous infusion of 4 Luminity vials (1.5 mL each, diluted with saline 0.9% to 40 mL total) of microbubbles with the use of local ultrasound at the site of occlusion. Primary endpoints are the incidence of complications and technical feasibility. Secondary endpoints are angiographic and clinical success, duration of thrombolytic infusion, treatment-related mortality, amputations, additional interventions, and quality of life.

Ethics and dissemination

Ethical approval for this study was obtained in 2015 from the Medical Ethics Committee (METC) of the VU University Medical Center, Amsterdam, the Netherlands. A statement of consent for this study was given by the Dutch national competent authority. Data will be presented at national and international conferences and published in a peer-reviewed journal.

Registration: Dutch National Trial Registry: NTR4731; European Clinical Trials Database (EudraCT) of the European Medicines Agency: 2014-003469-10.

For peer review only

STRENGTHS AND LIMITATIONS

- This will be a first in man study to examine the safety and technical feasibility of therapeutic microbubbles, combined with the application of ultrasound and catheter-directed thrombolysis in peripheral arterial occlusions.

- This is a 'single arm' trial. The data will be used to inform a future large multicentre randomised controlled trial comparing conventional catheter-directed thrombolysis with microbubble and ultrasound accelerated thrombolysis

- **The present study is a non-randomized Phase II-trial, therefore the results cannot confirm benefit of sonothrombolysis for peripheral arterial occlusions, only safety and feasibility is analyzed.**

- **The present study does not compare other thrombolysis techniques or protocols.**

For peer review only

For peer review only - http://bmjopen.bmj.com/site/about/guidelines.xhtml

INTRODUCTION

Acute limb ischemia can be caused by a thrombus occluding an artery in an arm or leg. This is an emergency situation that can result in amputation or death if not treated successfully.[1] Intra-arterial infusion of thrombolytic agents, i.e. catheter-directed thrombolysis, can restore blood flow by dissolving the clot, as a less invasive alternative to surgical thromboembolectomy.[2] In comparison with the lysis of small arterial occlusions in patients with myocardial infarction, larger peripheral arterial occlusions require higher doses of lytic agents and infusion over a longer period of time. Inevitably, such treatment is accompanied by a risk of major hemorrhagic complications, such as hemorrhagic stroke, in up to 8% of patients.[3] Furthermore, this technique is time-consuming (several days of bed rest is usually required) and repeated angiography for treatment monitoring is needed, putting patients at risk for contrast-induced nephropathy. As a result, this leads to high morbidity rates and a significant patient burden. Methods to improve this therapy are therefore highly sought after.

A potential accelerator of thrombolytic therapy is contrast-enhanced ultrasound. Contrast agents, consisting of 5-10 μm gas-filled particles (microbubbles), have initially been used as diagnostic ultrasound contrast-enhancers. A new field of research investigates these agents for potential therapeutic purposes such as targeted drug delivery and thrombolysis.[4] The proposed mechanism of action in thrombolysis is that microbubbles can oscillate under the influence of ultrasound. At high intensities, this oscillation can lead to microbubble collapse and the production of mechanical forces on the clot surface, making the thrombus more susceptible to thrombolytics, thus accelerating thrombolysis.[5]

In early stages of clinical research, this technique has been shown to be efficient as treatment for acute cerebral stroke and acute myocardial.[6,7] Although the safety of their clinical administration in treating smaller arteries in the heart has been a topic of discussion in the past, post-marketing data for diagnostic indications showed continued safety after extensive research in more recent years.[8,9,10] For therapeutic thrombolytic purposes, this technique has been shown to be effective and safe in a porcine model of large peripheral arterial occlusions.[11] In this study, we will investigate the therapeutic application of microbubbles with ultrasound in combination with catheter-directed thrombolysis for patients with peripheral arterial occlusions. An illustrative video regarding our research project is available at https://vimeo.com/159929945/4aaf6770cf.

For peer review only

METHODS AND ANALYSIS

Study objectives

To investigate the safety and practical applicability of the therapeutic application of microbubbles and ultrasound in combination with catheter-directed thrombolysis for patients with peripheral arterial occlusions.

Design

The Microbubbles and UltraSound accelerated Thrombolysis (MUST) trial is a single-arm phase-II trial.

Primary study parameters

Main endpoints will be the safety and technical feasibility of the experimental treatment. Safety will be determined by treatment-related mortality, the occurrence of Adverse Events (AEs), Serious Adverse Events (SAEs) and Suspected Unexpected Serious Adverse Reactions (SUSARs). AEs will be defined as any undesirable experience occurring to a subject during the experimental treatment period, whether or not considered to be related to the investigational drug or intervention. SAEs will be defined as any untoward medical occurrence or effect that at any dose results in death; is life threatening (at the time of the event); requires hospitalization or prolongation of existing in-patients' hospitalization; results in persistent or significant disability or incapacity; is a new event of the trial likely to affect the safety of the subjects, such as an unexpected outcome of an adverse reaction. SUSARs, which are related to the microbubble infusion and ultrasound application, are the formation of micro embolisms resulting in occlusion of the microcirculation, hemorrhages, hypotension, heart rhythm disorders and anaphylaxis. See the paragraph *adverse events* for detailed handling procedures of AEs, SAEs and SUSARs. Hemorrhagic complications related to thrombolytic therapy will be reported according the Standardized Bleeding Definitions for Cardiovascular Clinical Trials proposed by Mehran et al.[12]
Technical feasibility will be defined as accomplishment of the experimental protocol during the first hour of thrombolysis.

Secondary study parameters

Angiographic success will be defined as dissolution of >95% of the thrombus with outflow to at least 1 crural artery. Clinical change/success will be reported according to Rutherford's recommended scale for gauging changes in clinical status (Table I). Amputations will be defined as either major (above or below knee amputation) or minor (metatarsal or toe amputation). Additional interventions will be categorized as either surgical (for example thromboembolectomy, bypass graft surgery) or percutaneous (Percutaneous Transluminal Angioplasty, stent placement) and as either required for restoration of patency or necessary for correction of underlying lesions. We will also determine microcirculation of the limb (by Laser Doppler measurements, Perimed Instruments, Järfälla, Sweden), 30-day mortality, conversion to surgery, serum fibrinogen concentrations measured during thrombolytic treatment on a daily basis, pain by Visual Analogue Scale (VAS) and quality of life by SF-36 questionnaires. The duration of thrombolysis will be defined by the time-span between initiation and completion angiography.

Table I: *Rutherford's recommended scale for gauging changes in clinical status*

Adapted from: Rutherford RB, Baker JD, Ernst C, Johnston KW, Porter JM, Ahn S, et al. Recommended standards for reports dealing with lower extremity ischemia: revised version. J Vasc Surg. 1997;26(3):517–38.

+3	Markedly improved: No ischemic symptoms, and any foot lesions completely healed; ABI essentially "normalized" (increased to more than 0.90)
+2	Moderately improved: No open foot lesions; still symptomatic but only with exercise and improved by at least one clinical chronic ischemia category; ABI not normalized but increased by more than 0.10
+1	Minimally improved: Greater than 0.10 increase in ABI* but no categorical improvement or vice versa (i.e., upward categorical shift without an increase in ABI of more than 0.10)
0	No change: No categorical shift and less than 0.10 change in ABI

For peer review only - http://bmjopen.bmj.com/site/about/guidelines.xhtml

−1	Mildly worse: No categorical shift but ABI decreased more than 0.10, or downward categorical shift with ABI decrease less than 0.10
−2	Moderately worse: One category worse or unexpected minor amputation
−3	Markedly worse: More than one category worse or unexpected major amputations
*In cases where the ABI cannot be accurately measured, an index based on the toe pressure, or any measurable pressure distal to the site of revascularization, may be substituted.	

Abbreviations: *ABI=Ankle Brachial Index*

For peer review only

Patients and eligibility criteria

The present feasibility and safety study is a non-randomized Phase II trial, to be conducted in our university hospital in Amsterdam, the Netherlands. Usually in a phase-II trial, 10-20 patients are investigated to confirm an occurrence of toxic effects or serious adverse events <20%. We chose a sample size of 20 to assess the safety of the investigational treatment. Eligibility criteria are listed in Table II. Inclusion of 20 eligible patient is expected within 1.5 years. Written informed consent will be acquired by a member of the Research Team after information about the study has been provided by the treating doctor.

Table II: *Eligibility criteria*

Inclusion criteria	• Men and women older than 18 and younger than 85 years old<br>• Patients with a maximum of 2 weeks of symptoms for lower limb ischemia due to thrombosed/occluded iliofemoral, femoropopliteal or femorocrural native arteries or iliofemoral, femoropopliteal or femorocrural venous or prosthetic bypass grafts<br>• Patients appropriate for thrombolysis i.e. with acute lower limb ischemia class I and IIa according to the Rutherford classification<br>• Patients who understand the nature of the procedure and provide written informed consent before enrollment in the study
Exclusion criteria	• Patients with clinical complaints of acute lower limb ischemia due to thrombosis of iliofemoral, femoropopliteal or femorocrural native arteries, or iliofemoral, femoropopliteal or femorocrural venous or prosthetic bypass grafts for more than 2 weeks<br>• Patients with thrombosed popliteal aneurysms<br>• Patients with absolute contraindications for administration of antiplatelet therapy, anticoagulants or thrombolytics<br>• History of recent (less than 6 weeks) ischemic stroke, cerebral hemorrhagic or myocardial infarction<br>• Patients with recent (less than 6 weeks) surgery<br>• Severe hypertension (diastolic blood pressure greater than 110 mm Hg, systolic blood pressure higher than 200 mm Hg)<br>• Current malignancy or severe co-morbid condition with a life expectancy of less than 6 months<br>• Patients with uncorrected bleeding disorders (gastrointestinal ulcer, menorrhagia, liver failure)<br>• Women with childbearing potential not taking adequate contraceptives or currently breastfeeding<br>• Pregnancy<br>• Patients who are currently participating in another investigational drug or device study<br>• Patients younger than 18 years or older than 85 years<br>• Patients with contraindications for Luminity microbubbles i.e.:<br>○ Hypersensitivity to perflutren or to any of the components of Luminity

	○ Recent acute coronary syndrome or clinically unstable ischemic cardiacdisease, including: evolving or ongoing myocardial infarction, unstable angina at rest within the last 7 days, significant worsening of cardiac symptoms within the last 7 days, recent coronary artery intervention or other factors suggesting clinical instability (for example, recent deterioration of ECG, laboratory or clinical findings), acute cardiac failure, Class III/IV cardiac failure, or severe rhythm disorders ○ Patients known to have right-to-left cardiac shunts, severe pulmonary hypertension (pulmonary artery pressure >90 mmHg), uncontrolled systemic hypertension and in patients with GOLD Stage IV COPD, diffuse interstitial fibrosis or adult respiratory distress syndrome<br>○ Patients with cardiovascular instability where dobutamine is contraindicated

Data handling

We will keep an electronic log of patients who fulfill the eligibility criteria, patients who are invited to participate in the study, patients recruited and patients who withdraw from the study. Reasons for non-recruitment will also be recorded. We will attempt to collect reasons for non-participation from patients who decline to take part. During the course of the study, we will document reasons for withdrawal from the study and loss to follow-up. Data will be stored electronically in Case Report Forms software with audit trail functionality and will be audited by the institutional Clinical Research Bureau (CRB). Only anonymized information will be stored and participants will only be identifiable by their unique study number, which will be kept in a separate file. Data will be securely stored on these servers for 15 years according to national guidelines. The principal investigator will have access to the final trial dataset. No independent Data Management Committee was instated according to local ethics committee guidelines since the present study was not classified as a high-risk clinical study. This classification was approved by the local ethics committee based on the risk assessment form of the Netherlands Federation of University Medical Centres.

Study procedures

Intervention

A flow chart of the patient work-up after presentation in our hospital is presented in Figure 1. Low-dose thrombolytic treatment with urokinase will be initiated following our standard institutional protocol: a catheter is placed intra-arterially in the affected artery and a bolus injection of 500,000 International Units (IU) of urokinase (Medacinase Urokinase, Medac GmbH, Hamburg, Germany) will be followed by the continuous infusion of 50,000 units of UK per hour and 9,600 IU of heparin per 24 hours. The experimental treatment consists of (in addition to the standard thrombolytic therapy) the use of local 1.8 Mhz transdermal ultrasound (Philips iE33 Ultrasound Machine, Eindhoven, the Netherlands), and the intravenous infusion of 4 Luminity vials (total 6 mL, diluted with saline 0.9% to 40 mL total, Lantheus MI UK Limited, Newbury, Berkshire, United Kingdom) during the first hour of thrombolysis with urokinase. An ACIST VueJect (Bracco Imaging Europe B.V., the Netherlands) infusion pump will be used to infuse the 4 vials continuously. Ultrasound will be intermittently activated (3 seconds manual flash to burst microbubbles with Mechanical Index (MI) 1.08 (pulse duration 20 microseconds, frequency 1.8 Mhz, framerate 39 Hz), 7 seconds of visualization of inflow of the microbubbles at MI±0.11, at the site of occlusion during the first hour of thrombolysis. Criteria for discontinuation of the experimental treatment during the first hour will be the occurrence of any adverse events potentially related to the experimental treatment such as bleeding and allergic reactions.

For peer review only

Figure 1: *Flow chart of patient work-up after presentation*

Assessments

Diagnostic measurements

Additional diagnostic measurements during admission including ECG, duplex ultrasound, angiography and microcirculation measurements (by Laser Doppler flowmetry) will be performed as depicted in Figure 1.

A duplex ultrasound will be performed every 6±2 hours to monitor for signs of revascularization. When resumption of flow is visualized by duplex ultrasound, angiography will be performed to confirm flow. Angiography will be performed at least once daily as standard procedure. Outside of routine hospital working hours, angiography will only be performed in emergencies as per standard care.

A standardized pain score (Visual Analogue Scale, 1-10), and Pain Catastrophizing Scale will be recorded every 3 hours by a nurse practitioner, research fellow or surgical resident to assess pain.

Fibrinogen monitoring

Following our standard institutional thrombolysis protocol, fibrinogen concentration will be checked during thrombolysis with the following criteria for treatment modification: If <1.0 g/L, the urokinase infusion rate will be lowered to 25,000 IU/h; if <0.5 g/L, thrombolysis must be aborted temporarily and replaced by normal saline infusion. Three hours following treatment modifications, fibrinogen concentration will be reevaluated and when >1.0 g/L thrombolysis will be restarted at an initial low dose urokinase of 50,000 IU/h.

Post-procedural anticoagulation

After successful thrombolysis, the patient will be heparinized with low-molecular weight heparin (fraxiparine) dosed based on body weight: <50 kg: 2 times a day 3,800 IU (= 0.4 mL), 50-80 kg: 2 times a day 5,700 IU (= 0.6 mL), >80 kg: 2 times a day 7,600 IU (= 0.8 mL).

Concomitant therapy with coumarin derivatives will also be started at that time. Activated partial thromboplastin time (aPTT) will be measured daily during heparin treatment. The target range international normalized ratio (INR) will be 2.5 to 3.5; if this value is reached, heparinization will be stopped and coumarin treatment will be continued.

Follow-up

Outpatient follow-up will take place at specific time points for a total duration of 1 year, measurements performed during follow-up visits are depicted in Figure 1.

Adverse events

Adverse events will be recorded in detail in the electronic patient record. Any serious adverse events that occur after joining the trial will be reported to the accredited Medical Ethics Committee of our institution according to national and institutional guidelines. All adverse events will be followed up until they have abated, or until a stable situation has been reached. Depending on the event, follow up may require additional tests or medical procedures as indicated, and/or referral to a general physician or medical specialist. An interim analysis after 10 patients will be performed and if serious adverse events have occurred, we will discuss the continuation of the study. The study will be prematurely terminated if 2 or more intracranial bleedings occur or more than 5 allergic reactions.

Statistical analysis

For peer review only

Categorized epidemiologic/descriptive patient variables are summarized with frequencies and will be analyzed with Fischer's exact test or the Pearson Chi-squared test. To avoid possible violations of the assumptions for parametric testing, such as a normal distribution pattern, we will employ non-parametric methods such as a Spearman rank correlation and a Mann-Whitney U test in the case of a skewed distribution or log transformation. For associations of two outcome measurements, we will use a correlation (Spearman rank) or single regression analysis. We will analyze the following outcomes by means of Kaplan-Meier curves: patency rate, amputation-free rate, and intervention-free rate. We will assess heterogeneity in prognostic factors as a secondary analysis by means of Chi-squared tests. All tests will be performed two-sided, and a $p<0.05$ will be considered to be statistically significant.

ETHICS AND DISSEMINATION

The study will be conducted according to the principles of the Declaration of Helsinki (Brazil, October 2013), and in accordance with the Medical Research Involving Human Subjects Act (WMO). An Investigator Site File will be produced in advance of the study conforming to institutional guidelines. Furthermore we will create Case Report Forms by using GCP and 21 CFR Part 11 compliant software, to handle patient data.

The study has been registered in the Dutch Trial Register, at the Dutch National Central Committee on Research Involving Human Subjects (CCMO) and in the European Clinical Trials Database (EudraCT) of the European Medicines Agency. Any protocol amendments during the study will be communicated and changed accordingly in the relevant registries after approval of the institutional Medical Ethics Committee. The results of this study will be submitted for publication in a peer-reviewed journal, regardless of the outcome of this study, according to the CCMO statement on publication policy. Data will also be presented at international conferences.

For peer review only

For peer review only - http://bmjopen.bmj.com/site/about/guidelines.xhtml

DISCUSSION

The Microbubbles and UltraSound-accelerated Thrombolysis (MUST) trial is a phase-II single-arm clinical trial. In this study the safety and feasibility of an experimental ultrasound technique will be investigated for the first time in patients with large peripheral arterial occlusions.

We believe that this procedure is safe and can accelerate thrombolysis, thereby allowing for reduction of thrombolytic dosage, which in turn reduces the risk of major hemorrhagic complications.

An experimental bolus therapy with microbubbles and ultrasound could accelerate thrombolysis because at high ultrasound intensities microbubbles can collapse, resulting in mechanical forces on the clot surface. The formation of small channels in the thrombus lead to exposure of a larger total surface susceptible to thrombolytics.[5]

In regard to the therapeutic application of contrast agents, several studies have been performed in patients with ischemic stroke and myocardial infarction. A systematic review of sonothrombolysis shows that this treatment option improves clinical and long-term outcomes, while potentially reducing bleeding risk, in patients with ischemic stroke.[13] Nevertheless, dose escalation studies show that the safety (in terms of bleeding and micro emboli) needs to be further investigated before enrolling patients in phase-III trials.[14] Few and heterogeneous studies examined the therapeutic application of sonothrombolysis in patients with myocardial infarction. Although pilot studies affirm safety and feasibility, the application of therapeutic ultrasound with longer pulse durations (20 microseconds vs. 5 microseconds) was reported to result in unexpected coronary vasoconstriction in a recent clinical trial.[15]
Potential reported mechanisms for this effect are the summative effect of myocardial ischemia, reperfusion damage and long-pulse-duration sonoporation on endothelial damage, all leading to calcium overload. However, patients with peripheral arterial occlusions are mostly chronic vascular patients who often have received previous treatments in the respective artery, for example thrombolytic therapy, percutaneous transluminal angioplasty, thromboembolectomy or bypass surgery. The mechanical manipulation of the vascular wall during all these treatments is extensive. Furthermore, during standard thrombolytic treatment, arteries are manipulated and perforated on purpose to insert guide wires and catheters. Hence, vascular spasms during these peripheral treatments are normal and non-threatening to the patient, in contrast to spasms in small coronary arteries.

The administration of ultrasound contrast agents has been accompanied by important discussions regarding safety concerns in the past[8,16]. As a response to the occurrence of SAEs, the US Food and Drug Administration (FDA) issued a labeling change and warnings for contrast agents in 2007. Consequently, new studies on the risks of contrast agents were performed and these established their safety.[17]

In regard to Luminity contrast agent dose regimens, the recommended dose for diagnostic indications is 1.3 mL dispersion added to 50 mL of sodium chloride 9 mg/mL (0.9%) or glucose 50 mg/mL (5%) solution injected over a short time-period.[18] For therapeutic purposes, in large peripheral arteries there are no dose studies available. However, in our university hospital the Sonolysis study has been performed by our Cardiology department to treat acute ST elevation myocardial infarction patients with Luminity microbubbles and high mechanical ultrasound.[19] The dose used was one flacon Luminity of 1.5 mL which contains 225 microliter perflutren diluted with 48.5 mL of saline 0.9% to create a 50 mL suspension. Patients were treated with 15 minutes with an infusion rate of 200 mL/h. No serious adverse events occurred. In the present study to establish a therapeutic effect in large arterial occlusion we will also infuse 1 vial per 15 minutes but we will treat patients for 60 minutes. We will use 4 flacons of 1.5 mL Luminity containing 900 microliter perflutren diluted with saline 0.9% to 40 mL total volume to be infused during 1 hour. The clinical consequences of overdose with Luminity are not known. Single doses of up to 100 microlitres dispersion/kg and multiple doses up to 150 microlitres dispersion/kg were tolerated well in Phase I clinical trials.[20] This equals to the infusion of 12 mL (8 flacons) of Luminity dispersion. We will administer a total of 6 mL (4 flacons) of Luminity dispersion. Furthermore, we will not administer them as single bolus doses but as low-speed continuous infusion. During the experimental protocol with microbubble infusion, patients will be continuously monitored.

For peer review only - http://bmjopen.bmj.com/site/about/guidelines.xhtml

For peer review only

As with all contrast agents, the risk of anaphylactic reactions to contrast remains. Therefore, administration of contrast agents in a center with full resuscitation possibilities is mandatory. Furthermore, during the first hours of administration, monitoring of vital parameters of patients is important.

In this study, thrombolysis is performed with the fibrinolytic urokinase, which is the most used fibrinolytic agent for the treatment of peripheral arterial occlusions worldwide and is standard care in the Netherlands. Some countries use tissue plasminogen activator for this indication. A Cochrane review on the topic states that there is no evidence that (r)t-PA is more effective than urokinase for patients with peripheral arterial occlusion.[21]

If the application of microbubbles and ultrasound concomitant to catheter-directed thrombolysis is shown to be safe and technically feasible based on this phase-II trial, we anticipate a funding application for a larger randomized controlled trial with a comparative group to assess and compare efficacy of this treatment.

Although the efficacy of the currently described protocol cannot be adequately compared within this study design, we will discuss the outcomes relative to a historic control group that had previously received our standard hospital thrombolysis protocol.[22]

Successful thrombolysis is strongly predictive of amputation-free survival with vascular patency for at least one year.[23] A longer duration of thrombolysis inevitably exposes a patient to a higher thrombolytic dose and higher risk of hemorrhage, in addition to an already increased patient burden because of prolonged bed rest. Therefore ultimately, acceleration of thrombolysis with microbubbles could benefit the patient because of a shorter therapy time, a lower risk of hemorrhagic complications and a decrease in patient burden.

ACKNOWLEDGEMENTS:
We would like to thank Laura van Wieringen, Lloyd Belliot, Joyce Lu, Jorn Meekel, Ted van Schaik and Sean Matheiken for their assistance in preparation of this study.

AUTHOR CONTRIBUTIONS

HE, JN, WW and KK planned and designed the research, HE wrote the manuscript, HE, JN, RL and KK critically revised the manuscript and all authors and MUST collaborators approved the final version of the manuscript.

FUNDING: This study is supported by the Departments of Surgery, Radiology, Cardiology and Physiology of the VU University Medical Center Amsterdam, the Netherlands and by a unrestricted research grant from LamePro B.V., the Netherlands. LamePro B.V. has no role in the study design, collection, management, analysis, and interpretation of data, nor in writing of the report, submitting the report for publication or ultimate authority on any of the previous mentioned.

COMPETING INTERESTS: none

For peer review only - http://bmjopen.bmj.com/site/about/guidelines.xhtml

REFERENCES

1. Creager, M. A., Kaufman, J. A., & Conte, M. S. Clinical practice. Acute limb ischemia. *N Engl J Med*, 2012. 366(23), 2198–2206.
2. Berridge, D. C., Kessel, D., & Robertson, I. Surgery versus thrombolysis for acute limb ischaemia: initial management. *Cochrane Database Syst Rev*, (3) (2013), CD002784.
3. van den Berg, J. C. Thrombolysis for acute arterial occlusion. *J Vasc Surg*, 2009. 52(2), 512–515.
4. Dijkmans, P. A., Juffermans, L. J. M., Musters, R. J. P., Wamel, A. Van, Cate, F. J., Gilst, W. Van, Kamp, O. Microbubbles and ultrasound: from diagnosis to therapy. *Eur J Echocardiogr.* 2004 Aug;5(4):245-56.
5. Everbach, E. C., & Francis, C. W. (2000). Cavitational mechanisms in ultrasound-accelerated thrombolysis at 1 MHz. *Ultrasound Med Biol*, 2000 26(7), 1153–1160.
6. Balucani C, Alexandrov A V. Ultrasound- and microspheres-enhanced thrombolysis for stroke treatment: state of the art. *Curr Cardiol Rep*. 2010;12(1):34–41.
7. Slikkerveer J, Kleijn SA, Appelman Y, Porter TR, Veen G, van Rossum AC, et al. Ultrasound enhanced prehospital thrombolysis using microbubbles infusion in patients with acute ST elevation myocardial infarction: pilot of the Sonolysis study. *Ultrasound Med Biol*. 2012;38(2):247–52.
8. Dijkmans PA, Visser CA, Kamp O. Adverse reactions to ultrasound contrast agents: Is the risk worth the benefit? *Eur J Echocardiogr*. 2005 *Oct;6(5):363-6.*
9. Roos ST, Juffermans LJM, van Royen N, van Rossum AC, Xie F, Appelman Y, et al. Unexpected High Incidence of Coronary Vasoconstriction in the Reduction of Microvascular Injury Using Sonolysis (ROMIUS) Trial. *Ultrasound Med Biol*. 2016 May 5 (Epub ahead of print).
10. Appis a. W, Tracy MJ, Feinstein SB. Update on the safety and efficacy of commercial ultrasound contrast agents in cardiac applications. Echo Res Pract. 2015;2(2):R55–62.
11. Ebben HP, Nederhoed JH, Slikkerveer J, Kamp O, Tangelder GWJM, Musters RJP, et al. Therapeutic application of contrast-enhanced ultrasound and low-dose urokinase for thrombolysis in a porcine model of acute peripheral arterial occlusion. *J Vasc Surg*. 2015 Aug;62(2):477–85.
12. Mehran R, Rao S V., Bhatt DL, Gibson CM, Caixeta A, Eikelboom J, et al. Standardized Bleeding Definitions for Cardiovascular Clinical Trials: A Consensus Report From the Bleeding Academic Research Consortium. *Circulation*. 2011 Jun 13;123(23):2736–47.
13. Roos ST, Juffermans LJM, Slikkerveer J, Unger EC, Porter TR, Kamp O. Sonothrombolysis in acute stroke and myocardial infarction: A systematic review. *IJC Hear Vessel*.; 2014;4:1–6.
14. Molina CA, Barreto AD, Tsivgoulis G, Sierzenski P, Malkoff MD, Rubiera M, et al. Transcranial ultrasound in clinical sonothrombolysis (TUCSON) trial. *Ann Neurol*.; 2009;66(1):28–38.
15. Roos ST, Juffermans LJM, van Royen N, van Rossum AC, Xie F, Appelman Y, et al. Unexpected High Incidence of Coronary Vasoconstriction in the Reduction of Microvascular Injury Using Sonolysis (ROMIUS) Trial. *Ultrasound Med Biol* [Internet]. 2016;1–10.
16. Douglas PS, Weyman AE, Lindner JR, Wei K.Contrast echocardiography: past, present, and...future? *JACC Cardiovasc Imaging.* 2008 Jan;1(1):107-10.
17. FDA Advisory Committee Briefing Document Cardiovascular and Renal Drugs Advisory Committee June 24th 2008. (Accessed on 23-05-2016, available at http://www.fda.gov/ohrms/dockets/ac/08/briefing/2008- 4369b1-01.pdf).
18. EMA Luminity Summary of Product Characteristics (SmPC) (available at http://www.ema.europa.eu/docs/en_GB/document_library/EPAR_-_Product_Information/human/000654/WC500045020.pdf)

19. Slikkerveer J, Kleijn SA, Appelman Y, Porter TR, Veen G, van Rossum AC, et al. Ultrasound enhanced prehospital thrombolysis using microbubbles infusion in patients with acute ST elevation myocardial infarction: pilot of the Sonolysis study. Ultrasound Med Biol. England; 2012;38(2):247–52.
20. Luminity: EPAR - Scientific Discussion (available at http://www.ema.europa.eu/docs/en_GB/document_library/EPAR_-_Scientific_Discussion/human/000654/WC500045016.pdf)
21. Robertson I, Kessel DO, Berridge DC. Fibrinolytic agents for peripheral arterial occlusion. *Cochrane Database Syst Rev*. 2013 Dec 19;12:CD001099.
22. Ebben HP, Nederhoed JH, Lely RJ, Meijerink MR, van der Meijs BB, Wisselink W, et al. Low-dose Thrombolysis for Thromboembolic Lower Extremity Arterial Occlusions is Effective Without Major Hemorrhagic Complications. *Eur J Vasc Endovasc Surg*. 2014 Nov;48(5):551-8.
23. G. Plate, S. Oredsson, J. Lanke. When is thrombolysis for acute lower limb ischemia worthwhile? *Eur J Vasc Endovasc Surg*, 37 (2) (2009), pp. 206–212

For peer review only

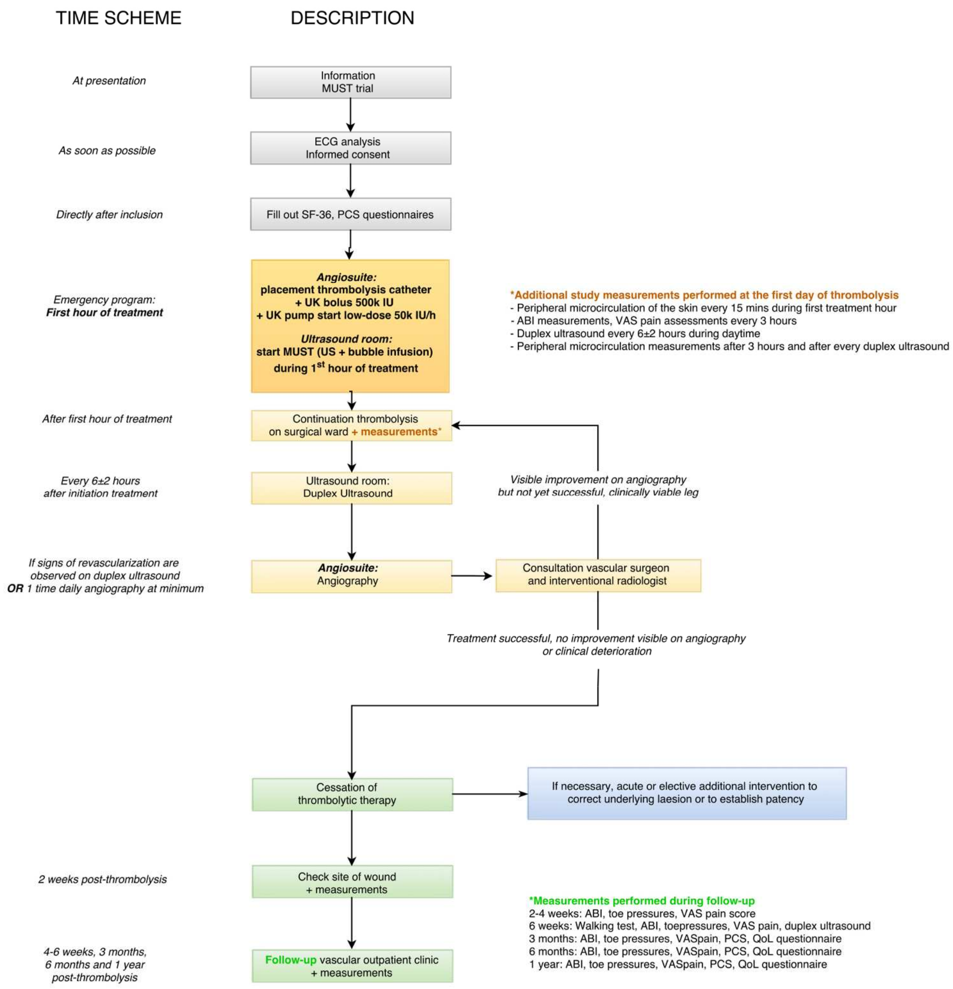

Flow chart of patient work-up after presentation.
Abbreviations: ECG = Electro Cardiography, UK = urokinase, US = UltraSound, ABI = Ankle Brachial Index, VAS = Visual Analogue Scale, PCS= Catastrophizing Scale, QoL=Quality of Life.

Figure 1
157x167mm (300 x 300 DPI)

Korte proefpersoneninformatiebrief

MUST Trial

Microbubbels en ultrageluid toegevoegd aan een stolseloplossend medicijn

Volledige Engelse titel:
The application of contrast enhanced ultrasound (CEUS) to facilitate thrombolysis in patients with acute peripheral arterial occlusions.

VU medisch centrum

For peer review

For peer review only - http://bmjopen.bmj.com/site/about/guidelines.xhtml

Geachte heer/mevrouw,

Wij vragen u vriendelijk om mee te doen aan een medisch-wetenschappelijk onderzoek waarin een nieuwe behandeling voor een vaatafsluiting in het been wordt onderzocht. U beslist zelf of u wilt meedoen.

In deze korte informatiebrief wordt u uitgelegd wat het onderzoek inhoudt. Na afloop van de behandeling ontvangt u een uitgebreide informatiebrief over het onderzoek.

Wat wordt er onderzocht en waarom?

In dit onderzoek wordt de behandeling met echo en contrast bovenop de standaard stolseloplossende behandeling onderzocht bij patiënten met een stolsel in hun beenvaten. Het doel is de trombolysebehandeling te versnellen met behulp van echogeluidsgolven en microbubbles. Microbubbles zijn kleine gasbelletjes die normaal als contrast bij echo worden gebruikt. Ze werden dus al vaker in patiënten gebruikt om de echobeelden te verduidelijken. Wij gebruiken de trilkrachten van de microbubbles. Zij kunnen namelijk trillen door de echogeluidsgolven en het stolsel in uw vat mogelijk sneller oplossen.

Het toedienen van contrast bij echo wordt al jaren veilig bij patiënten toegepast om betere beeldvorming te krijgen en dit contrast is niet belastend voor de nieren. De inzet van echo met contrast bij het oplossen van een stolsel wordt ook in patiënten met hart- en herseninfarcten al veel onderzocht.

Wat staat u te wachten als u deelneemt aan dit onderzoek?

Nadat u schriftelijk toestemming heeft gegeven om deel te nemen aan het onderzoek zal er een korte vragenlijst worden afgenomen (duur ongeveer 2 minuten) en zal de standaard stolseloplossende behandeling gestart worden. Informatie over de stolseloplossende behandeling wordt gegeven door de arts van de afdeling of de behandelend chirurg.

For peer review only

For peer review only - http://bmjopen.bmj.com/site/about/guidelines.xhtml

De experimentele behandeling bestaat uit de toevoeging van echo met contrast gedurende het eerste uur van de standaard behandeling. Hiervoor wordt via een infuus in uw arm gedurende een uur contrast toegediend. Gedurende 1 uur zal dit infuus inlopen en zal een radioloog of echolaborant tegelijkertijd op uw been echogolven afgeven. De rest van de behandeling is zoals de standaard stolseloplossende behandeling. Gedurende de rest van de opname en bij een aantal controlebezoeken op de poli-kliniek zullen extra metingen worden verricht zoals
metingen van de doorbloeding van de huid, bloeddruk metingen in arm en been en het afnemen van een pijnscore en korte vragenlijst.
In de volgende afbeelding staat schematisch weergegeven hoe het gehele behandelingstraject eruit ziet voor u. **Dikgedrukt zijn alle handelingen en metingen die extra zijn door deel te nemen aan dit onderzoek** ten opzichte van een normale

For peer review only

For peer review only - http://bmjopen.bmj.com/site/about/guidelines.xhtml

trombolysebehandeling.

Tijdsmoment	***Omschrijving***
Direct bij opname	**Informatie MUST trial**
Zo snel mogelijk	**Analyse hartfilmpje**<br>**Toestemmingsverklaring**
Direct na besluit tot deelname aan onderzoek	**Invullen vragenlijst**
Via spoed programma:<br>Eerste uur van de behandeling	- Radiologieruimte: plaatsing slangetje in beenvat voor trombolyse + start stolseloplossende medicatie<br>**- Echokamer: start MUST (echo + contrastmiddel)**
Na 1e uur van behandeling	Vervolg trombolyse behandeling op opnameafdeling
Elke 6 uur na start behandeling	**Echokamer:**<br>**Echo onderzoek been**
Als tekenen van verbetering worden gezien met echo<br>**OF** minimaal 1x per dag	CTA scan
Gebaseerd op resultaten CTA scan na overleg vaatchirurg en interventieradioloog.	Stoppen trombolyse behandeling
2 weken na ontslag	Wondcontrole op polikliniek en **metingen**
**4 weken,** 6 weken, 3 maanden, 6 maanden en 1 jaar na ontslag	Controle op polikliniek en **metingen**

Toelichting enkele termen:

Een **CTA scan** is een speciale CT scan waarbij met contrastmiddel naar de vaten gekeken kan worden. De A staat voor angiografie. Deze scan is onderdeel van de standaard trombolyse behandeling en hiermee wordt gekeken naar de doorgankelijkheid van de beenvaten. Het contrastmiddel dat hierbij gebruikt wordt is een ander

For peer review only

For peer review only - http://bmjopen.bmj.com/site/about/guidelines.xhtml

contrastmiddel dan gebruikt bij de echo tijdens het eerste uur van de behandeling waar onderzoek naar gedaan wordt. Het contrast gebruikt tijdens de CTA scan is mogelijk schadelijk voor de nieren. Afhankelijk van uw nierfunctie worden hier mogelijk speciale voorbereidingen voor gedaan zoals het geven van vocht voor en na de scan om dit te voorkomen.

De controles op de polikliniek worden ook gedaan bij de standaard trombolysebehandeling, na 4 weken is er alleen een extra controlemoment bij de patiënten die deelnemen aan het onderzoek. De metingen die bij u gedaan worden tijdens dit controlebezoek zijn:

- Meting bloeddruk (armen, enkels, tenen)
- Pijnscore

Tijdens de controle op de polikliniek na 6 weken zal er behalve bovenstaande metingen ook een looptest en een echo gemaakt worden.

Na 3 en 6 maanden en na 1 jaar zal er ook een vragenlijst op de iPad ingevuld moeten worden met vragen over pijn en de kwaliteit van leven.

Wat zijn mogelijke voor- en nadelen van deelname aan dit onderzoek?

Mogelijk heeft u voordeel van de behandeling omdat deze door de nieuwe techniek verkort wordt. Mogelijk is hierdoor ook het risico op bloedingscomplicaties gedurende de behandeling verminderd. Tevens kan het onderzoek voor de toekomst nuttige gegevens opleveren voor de behandeling.

Nadelen van deelname zijn dat er gedurende het eerste uur van behandeling meer handelingen worden verricht en dat er gedurende de opname meer metingen worden verricht bij u. Tevens moet u voor en na behandeling bij terugkomst op de poli enkele malen een korte vragenlijst invullen. De mogelijke bijwerkingen van het echo contrast middel zijn in het algemeen niet ernstig en van voorbijgaande aard. De meest gemelde bijwerkingen zijn hoofdpijn (2%), pijn op de plaats van injectie (1%), reacties op de plaats van injectie waaronder bloeduitstortingen, warmte, verminderde gevoelswaarneming (2%). In enkele gevallen is een allergische reactie, verandering op het hartfilmpje (hier merkt u zelf meestal niets van), bloeddruk en in sommige

For peer review only - http://bmjopen.bmj.com/site/about/guidelines.xhtml

laboratoriumwaarden waargenomen (tijdelijke toename van witte bloedcellen, hier merkt u zelf meestal niets van).

Mocht u vragen hebben over het onderzoek of de behandeling dan kunt u terecht bij uw behandelend arts of een van de onderzoekers.

Met vriendelijke groet,

J.H. Nederhoed (vaatchirurg en onderzoeker), K.K. Yeung (chirurg in opleiding en coördinerend onderzoeker), H.P. Ebben (onderzoeker)

Mede namens,

Vaatchirurgen: H.M.E. Coveliers, A.W.J. Hoksbergen, J.A. Rauwerda, J.D. Blankensteijn, W. Wisselink
Interventieradioloog: R.J. Lely

Toestemmingsformulier

(Voor volwassenen die zelfstandig beslissingen kunnen nemen (wilsbekwaam zijn).)

Microbubbles en ultrageluid bij trombolyse

For peer review only

For peer review only - http://bmjopen.bmj.com/site/about/guidelines.xhtml

NL46636.029.14 18-11-2014

Ik heb de informatiebrief voor de proefpersoon gelezen. Ik kon aanvullende vragen stellen. Mijn vragen zijn genoeg beantwoord. Ik had genoeg tijd om te beslissen of ik meedoe.

Ik weet dat meedoen helemaal vrijwillig is. Ik weet dat ik op ieder moment kan beslissen om toch niet mee te doen. Daarvoor hoef ik geen reden te geven.

Ik geef toestemming om mijn huisarts te vertellen dat ik meedoe aan dit onderzoek. (indien van toepassing)

Ik geef toestemming om de specialist(en) die mij behandelt te vertellen dat ik meedoe aan dit onderzoek. (indien van toepassing)

Ik weet dat sommige mensen mijn gegevens kunnen zien. Die mensen staan vermeld in de Algemene brochure.

Ik geef toestemming om mijn gegevens te gebruiken, voor de doelen die in de informatiebrief staan.

Ik geef toestemming om mijn onderzoeksgegevens 15 jaar na afloop van dit onderzoek te bewaren.

Ik geef wel/geen* toestemming om mijn lichaamsmateriaal 15 jaar na afloop van dit onderzoek te bewaren, zodat dit in de toekomst misschien kan worden gebruikt voor onderzoek zoals beschreven in de informatiebrief. (indien van toepassing).

Ik wil meedoen aan dit onderzoek.

For peer review only

For peer review only - http://bmjopen.bmj.com/site/about/guidelines.xhtml

Naam proefpersoon:

Handtekening: Datum: __ / __ / __

Ik verklaar hierbij dat ik deze proefpersoon volledig heb geïnformeerd over het genoemde onderzoek.

Als er tijdens het onderzoek informatie bekend wordt die de toestemming van de proefpersoon zou kunnen beïnvloeden, dan breng ik hem/haar daarvan tijdig op de hoogte.

Naam onderzoeker (of diens vertegenwoordiger):

Handtekening: Datum: __ / __ / __

Aanvullende informatie is gegeven door (indien van toepassing):

Naam:

Functie:

Handtekening: Datum: __ / __ / __

----

* Doorhalen wat niet van toepassing is.

For peer review only

For peer review only - http://bmjopen.bmj.com/site/about/guidelines.xhtml

STANDARD PROTOCOL ITEMS: RECOMMENDATIONS FOR INTERVENTIONAL TRIALS

SPIRIT 2013 Checklist: Recommended items to address in a clinical trial protocol and related documents*

BOLD AND ITALIC TEXT MEANS CHECKED (manuscript page number for reference)

Section/item	Item No	Description
**Administrative information**		
Title	***1***	***Descriptive title identifying the study design, population, interventions, and, if applicable, trial acronym (page 1)***
Trial registration	***2a***	***Trial identifier and registry name. If not yet registered, name of intended registry (page 2)***
	2b	***All items from the World Health Organization Trial Registration Data Set (page 2)***
Protocol version	***3***	***Date and version identifier (page 1)***
Funding	***4***	***Sources and types of financial, material, and other support (page 12)***
Roles and responsibilities	***5a***	***Names, affiliations, and roles of protocol contributors (page 12)***
	5b	***Name and contact information for the trial sponsor (page 1)***
	5c	***Role of study sponsor and funders, if any, in study design; collection, management, analysis, and interpretation of data; writing of the report; and the decision to submit the report for publication, including whether they will have ultimate authority over any of these activities (page 12)***
	5d	***Composition, roles, and responsibilities of the coordinating centre, steering committee, endpoint adjudication committee, data management team, and other individuals or groups overseeing the trial, if applicable (see Item 21a for data monitoring committee) (NOT APPLICABLE (NA))***
**Introduction**		
Background and rationale	***6a***	***Description of research question and justification for undertaking the trial, including summary of relevant studies (published and unpublished) examining benefits and harms for each intervention (page 4)***

For peer review only

	6b	*Explanation for choice of comparators → NOT APPLICABLE*
Objectives	*7*	*Specific objectives or hypotheses (page 5)*
Trial design	*8*	*Description of trial design including type of trial (eg, parallel group, crossover, factorial, single group), allocation ratio, and framework (eg, superiority, equivalence, noninferiority, exploratory) (page 5)*

Methods: Participants, interventions, and outcomes

Study setting	*9*	*Description of study settings (eg, community clinic, academic hospital) and list of countries where data will be collected. Reference to where list of study sites can be obtained (page 6)*
Eligibility criteria	*10*	*Inclusion and exclusion criteria for participants. If applicable, eligibility criteria for study centres and individuals who will perform the interventions (eg, surgeons, psychotherapists) (page 6)*
Interventions	*11a*	*Interventions for each group with sufficient detail to allow replication, including how and when they will be administered (pages 7,8)*
	11b	*Criteria for discontinuing or modifying allocated interventions for a given trial participant (eg, drug dose change in response to harms, participant request, or improving/worsening disease) (page 7)*
	11c	*Strategies to improve adherence to intervention protocols, and any procedures for monitoring adherence (eg, drug tablet return, laboratory tests) (NA)*
	11d	*Relevant concomitant care and interventions that are permitted or prohibited during the trial (page 8)*
Outcomes	*12*	*Primary, secondary, and other outcomes, including the specific measurement variable (eg, systolic blood pressure), analysis metric (eg, change from baseline, final value, time to event), method of aggregation (eg, median, proportion), and time point for each outcome. Explanation of the clinical relevance of chosen efficacy and harm outcomes is strongly recommended (page 5)*
Participant timeline	*13*	*Time schedule of enrolment, interventions (including any run-ins and washouts), assessments, and visits for participants. A schematic diagram is highly recommended (see Figure) (page 8)*
Sample size	*14*	*Estimated number of participants needed to achieve study objectives and how it was determined, including clinical and statistical assumptions supporting any sample size calculations (page 6)*

Recruitment	*15*	*Strategies for achieving adequate participant enrolment to reach target sample size (page 6)*

Methods: Assignment of interventions (for controlled trials)

Allocation:

Sequence generation	*16a*	*Method of generating the allocation sequence (eg, computer-generated random numbers), and list of any factors for stratification. To reduce predictability of a random sequence, details of any planned restriction (eg, blocking) should be provided in a separate document that is unavailable to those who enrol participants or assign interventions (NA)*
Allocation concealment mechanism	*16b*	*Mechanism of implementing the allocation sequence (eg, central telephone; sequentially numbered, opaque, sealed envelopes), describing any steps to conceal the sequence until interventions are assigned (NA)*
Implementation	*16c*	*Who will generate the allocation sequence, who will enrol participants, and who will assign participants to interventions (NA)*
Blinding (masking)	*17a*	*Who will be blinded after assignment to interventions (eg, trial participants, care providers, outcome assessors, data analysts), and how (NA)*
	17b	*If blinded, circumstances under which unblinding is permissible, and procedure for revealing a participant's allocated intervention during the trial (NA)*

Methods: Data collection, management, and analysis

Data collection methods	*18a*	*Plans for assessment and collection of outcome, baseline, and other trial data, including any related processes to promote data quality (eg, duplicate measurements, training of assessors) and a description of study instruments (eg, questionnaires, laboratory tests) along with their reliability and validity, if known. Reference to where data collection forms can be found, if not in the protocol (page 9)*
	18b	*Plans to promote participant retention and complete follow-up, including list of any outcome data to be collected for participants who discontinue or deviate from intervention protocols (pages 8,9)*
Data management	*19*	*Plans for data entry, coding, security, and storage, including any related processes to promote data quality (eg, double data entry; range checks for data values). Reference to where details of data management procedures can be found, if not in the protocol (page 7)*

Statistical methods	*20a*	*Statistical methods for analysing primary and secondary outcomes. Reference to where other details of the statistical analysis plan can be found, if not in the protocol (page 9)*
	20b	*Methods for any additional analyses (eg, subgroup and adjusted analyses) (NA)*
	20c	*Definition of analysis population relating to protocol non-adherence (eg, as randomised analysis), and any statistical methods to handle missing data (eg, multiple imputation) (page 9)*
Methods: Monitoring		
Data monitoring	*21a*	*Composition of data monitoring committee (DMC); summary of its role and reporting structure; statement of whether it is independent from the sponsor and competing interests; and reference to where further details about its charter can be found, if not in the protocol. Alternatively, an explanation of why a DMC is not needed (NA)*
	21b	*Description of any interim analyses and stopping guidelines, including who will have access to these interim results and make the final decision to terminate the trial (NA)*
Harms	22	Plans for collecting, assessing, reporting, and managing solicited and spontaneously reported adverse events and other unintended effects of trial interventions or trial conduct (page 9)
Auditing	*23*	*Frequency and procedures for auditing trial conduct, if any, and whether the process will be independent from investigators and the sponsor (page 7)*
Ethics and dissemination		
Research ethics approval	*24*	*Plans for seeking research ethics committee/institutional review board (REC/IRB) approval (page 2)*
Protocol amendments	*25*	*Plans for communicating important protocol modifications (eg, changes to eligibility criteria, outcomes, analyses) to relevant parties (eg, investigators, REC/IRBs, trial participants, trial registries, journals, regulators) (page 10)*
Consent or assent	*26a*	*Who will obtain informed consent or assent from potential trial participants or authorised surrogates, and how (see Item 32) (page 6)*
	26b	*Additional consent provisions for collection and use of participant data and biological specimens in ancillary studies, if applicable (NA)*

only

Confidentiality	***27***	***How personal information about potential and enrolled participants will be collected, shared, and maintained in order to protect confidentiality before, during, and after the trial (page 7)***
Declaration of interests	***28***	***Financial and other competing interests for principal investigators for the overall trial and each study site (page 12)***
Access to data	***29***	***Statement of who will have access to the final trial dataset, and disclosure of contractual agreements that limit such access for investigators (page 7)***
Ancillary and post-trial care	***30***	***Provisions, if any, for ancillary and post-trial care, and for compensation to those who suffer harm from trial participation (NA)***
Dissemination policy	***31a***	***Plans for investigators and sponsor to communicate trial results to participants, healthcare professionals, the public, and other relevant groups (eg, via publication, reporting in results databases, or other data sharing arrangements), including any publication restrictions (page 10)***
	31b	***Authorship eligibility guidelines and any intended use of professional writers (page 12)***
	31c	***Plans, if any, for granting public access to the full protocol, participant-level dataset, and statistical code (NA)***
**Appendices**		
Informed consent materials	***32***	***Model consent form and other related documentation given to participants and authorised surrogates (attached in appendices)***
Biological specimens	***33***	***Plans for collection, laboratory evaluation, and storage of biological specimens for genetic or molecular analysis in the current trial and for future use in ancillary studies, if applicable (NA)***

*It is strongly recommended that this checklist be read in conjunction with the SPIRIT 2013 Explanation & Elaboration for important clarification on the items. Amendments to the protocol should be tracked and dated. The SPIRIT checklist is copyrighted by the SPIRIT Group under the Creative Commons "Attribution-NonCommercial-NoDerivs 3.0 Unported" license.

For peer review only
